# A guanidine-rich regulatory oligodeoxynucleotide improves type-2 diabetes in obese mice by blocking T-cell differentiation

**DOI:** 10.1002/emmm.201201272

**Published:** 2012-10-02

**Authors:** Xiang Cheng, Jing Wang, Ni Xia, Xin-Xin Yan, Ting-Ting Tang, Han Chen, Hong-Jian Zhang, Juan Liu, Wen Kong, Sara Sjöberg, Eduardo Folco, Peter Libby, Yu-Hua Liao, Guo-Ping Shi

**Affiliations:** 1 Laboratory of Cardiovascular Immunology, Institute of Cardiology, Union Hospital, Tongji Medical College of Huazhong University of Science and Technology; Laboratory of Biological Targeted Therapy of the Ministry of EducationWuhan, P. R. China; 2 Department of Medicine, Brigham and Women's Hospital and Harvard Medical SchoolBoston, MA, USA; 3 Department of Endocrinology, Union Hospital, Tongji Medical College of Huazhong University of Science and TechnologyWuhan, P. R. China

**Keywords:** macrophage, obesity, regulatory oligodeoxynucleotide, T-cell differentiation, type-2 diabetes

## Abstract

T lymphocytes exhibit pro-inflammatory or anti-inflammatory activities in obesity and diabetes, depending on their subtypes. Guanidine-rich immunosuppressive oligodeoxynucleotides (ODNs) effectively control Th1/Th2-cell counterbalance. This study reveals a non-toxic regulatory ODN (ODNR01) that inhibits Th1- and Th17-cell polarization by binding to STAT1/3/4 and blocking their phosphorylation without affecting Th2 and regulatory T cells. ODNR01 improves glucose tolerance and insulin sensitivity in both diet-induced obese (DIO) and genetically generated obese (*ob/ob*) mice. Mechanistic studies show that ODNR01 suppresses Th1- and Th17-cell differentiation in white adipose tissue, thereby reducing macrophage accumulation and M1 macrophage inflammatory molecule expression without affecting M2 macrophages. While ODNR01 shows no effect on diabetes in lymphocyte-free Rag1-deficient DIO mice, it enhances glucose tolerance and insulin sensitivity in CD4^+^ T-cell-reconstituted Rag1-deficient DIO mice, suggesting its beneficial effect on insulin resistance is T-cell-dependent. Therefore, regulatory ODNR01 reduces obesity-associated insulin resistance through modulation of T-cell differentiation.

## INTRODUCTION

Obesity and diabetes involve chronic inflammation (Hotamisligil, 2006). Recent studies in humans and animals have demonstrated that in addition to adipocytes, white adipose tissue (WAT) in obese subjects contains macrophages, mast cells and several different T-cell subsets (Caspar-Bauguil et al, 2005; Liu et al, 2009; Lumeng et al, 2009; Weisberg et al, 2003). These pro-inflammatory cells populate the stromal vascular fraction (SVF) of fat tissue where they produce inflammatory cytokines, proteases, growth factors and chemokines that modulate numerous WAT functions (Lago et al, 2007). Upon encountering antigenic peptides from antigen-presenting cells (APCs), CD4^+^ T cells polarize into different lineages, including T helper 1 (Th1), Th2, Th17 and regulatory T cells (Treg), as defined by their pattern of functions and cytokine profiles (Zhu et al, 2010). Recent studies have demonstrated an imbalance between dominant Th1 responses and reduced Th2 or Treg responses in WAT from obese subjects (Feuerer et al, 2009; Nishimura et al, 2009; Winer et al, 2009a). Enhanced Th1 cells in WAT produce interferon-γ (IFN-γ) leading to increased recruitment of adipose tissue macrophages (ATMs) and a shift in the activation state of ATMs from a protective alternatively activated (M2) state to a classically activated (M1) pro-inflammatory phenotype (Feuerer et al, 2009; Nishimura et al, 2009; Rocha et al, 2008; Winer et al, 2009a).

DNA exerts complex effects on the immune system, depending on its sequence. Bacterial DNA or synthetic phosphorothioate oligodeoxynucleotides (ODNs) that contain unmethylated CpG dinucleotides can stimulate immune responses in both Toll-like receptor 9 (TLR9)-dependent and TLR9-independent pathways (Krieg, 2002; Krieg et al, 1995; Landrigan et al, 2011), leading to the activation of macrophages, dendritic cells as well as B cells and the differentiation of Th1 and cytotoxic T cells. This class of ODNs can stimulate innate immune responses to heighten host resistance to infectious microorganisms and tumours, and to augment antigen presentation and facilitate the development of adaptive immune responses. Several ongoing clinical trials have examined the efficacy of such ODNs among patients with allergies, autoimmune diseases and cancers (Fonseca and Kline, 2009; Manegold et al, 2008; Schmidt, 2007). Unlike such immunostimulatory ODNs, regulatory ODNs mimic the immunosuppressive activity of the repetitive TTAGGG motifs present in mammalian telomeres, and can block the production of pro-inflammatory and Th1 cytokines triggered by polyclonal activators and antigens (Shirota et al, 2004; Gursel et al, 2003). In contrast, regulatory ODNs act by selectively binding to STAT1 and STAT4, then blocking their subsequent phosphorylation—and these activities do not require binding to receptors such as TLR9 (Shirota et al, 2004, 2005). In animals, regulatory ODNs can effectively ameliorate several Th1-biased autoimmune diseases, such as experimental autoimmune encephalomyelitis (EAE), arthritis and atherosclerosis (Zeuner et al, 2002, 2003; Cheng et al, 2008).

This study generated and evaluated several guanidine-rich ODNs and identified one regulatory ODN—termed regulatory ODNR01—that suppressed Th1 and Th17 differentiation more effectively and selectively than did previously described immunosuppressive ODNs. ODNR01 bound selectively to STAT1/3/4 and blocked their phosphorylation, limiting Th1 and Th17-cell differentiation. Peritoneal administration of ODNR01, but not of non-specific ODN1612, to diet-induced obese (DIO) C57BL/6 mice or genetically generated obese diabetic (*ob/ob*) mice muted the manifestations of type-2 diabetes, which required T cells.

## RESULTS

### Role of ODNR01 in suppressing Th1 and Th17 differentiation

To develop novel immunosuppressive ODNs, we designed four regulatory ODNs by changing the deoxynucleotides preceding the poly(G) motif. We used the most effective known immunosuppressive, ODNA151 (Klinman et al, 2008; Shirota et al, 2005), as a positive control to examine whether these novel regulatory ODNs effectively regulated T-cell differentiation.

**Figure 1:**
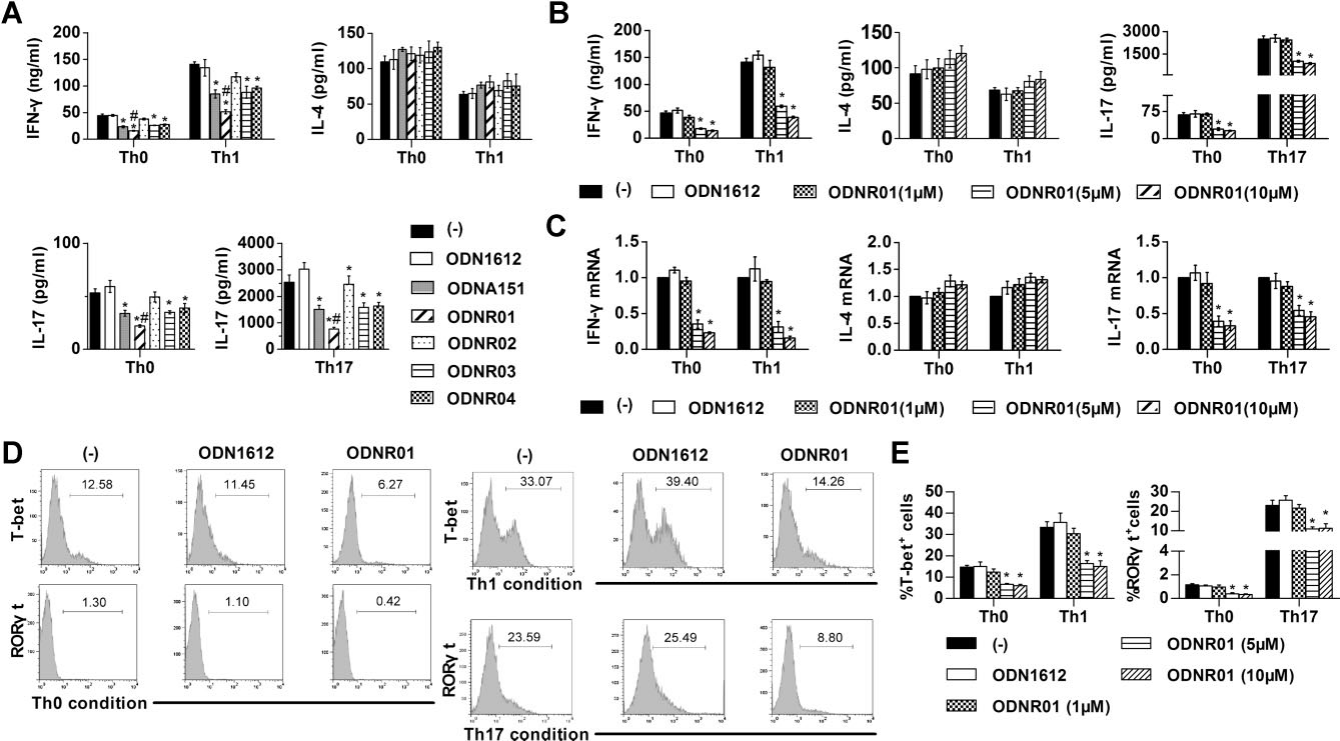
Immunosuppressive activity of ODNR01 on Th1- and Th17-cell differentiation CD4^+^ T-cell culture media IFN-γ, IL-4 and IL-17 levels as determined by ELISA. CD4^+^ T cells were treated with anti-CD3 and anti-CD28 mAb with or without (−) 5 µM of the different regulatory ODNs under Th0, Th1 or Th17 conditions for 3 days. Immunosuppressive ODNA151 was used as a positive control, and random ODN1612 was used as a negative control. *p < 0.05 *versus* (−) and ODN1612; #*p* < 0.05 *versus* ODNA151.ELISA determined CD4^+^ T-cell culture media IFN-γ, IL-4 and IL-17 levels after cells were cultured with or without ODN1612 (5 µM) and different doses of ODNR01 (1, 5 and 10 µM) under Th0, Th1 or Th17 conditions. *p < 0.05 *versus* (−) and ODN1612.RT-PCR determined CD4^+^ T-cell IFN-γ, IL-4 and IL-17 mRNA levels after cells were treated as in **B**. *p < 0.05 *versus* (−) and ODN1612.Representative FACS histograms of Th1 and Th17 cells for purified CD4^+^ T cells treated under Th0, Th1 or Th17 conditions with or without ODN (5 µM) for 4 days. Lymphocytes were first gated on the SSC/FSC plots and then the expression of T-bet and RORγt on the purified CD4^+^ T cells was analyzed.Th1- or Th17-cell frequencies in CD4^+^ T cells after cells were treated with ODNs as in **B** for 4 days, *p < 0.05 *versus* (−) and ODN1612. Data are representative of three independent experiments.

Peripheral CD4^+^ T cells from 8-week-old, Th1-biased C57BL/6 wild-type (WT) mice were cultured under different T-cell differentiation conditions (Th0, Th1 and Th17) in the presence of different ODNs (5 µM) as well as anti-mouse CD3 and anti-mouse CD28 monoclonal antibodies (mAbs). Enzyme-linked immunosorbent assay (ELISA) showed that, under Th0 conditions (no additives), three of four regulatory ODNs (ODNR01, ODNR03 and ODNR04) and ODNA151 significantly decreased IFN-γ and IL-17 production detectable in the culture media, but showed negligible effects in inhibiting IL-4 production. Among all effective ODNs, ODNR01 showed the most potent inhibition of IFN-γ and IL-17 production (Fig 1A). To explore further the effect of regulatory ODNs on T-cell differentiation, we repeated these studies in Th1 (IL-12 and anti-IL-4) and Th17 (IL-6, TGF-β, IL-23, anti-IFN-γ and anti-IL-4) culture conditions, which allowed Th1 and Th17 differentiation, respectively (Cui et al, 2009). Yet even under the Th1 and Th17 culture conditions, three regulatory ODNs and immunosuppressive ODNA151 significantly decreased IFN-γ (Th1) and IL-17 (Th17) production, and ODNR01 retained considerable inhibitory activities (Fig 1A). Under all conditions, the non-specific ODN1612 did not affect any of the cytokines tested.

Because regulatory ODNR01 comprising tandem TAGGG motifs showed the most immunosuppressive ability on Th1 and Th17 differentiation among all four novel regulatory ODNs and the known immunosuppressive ODNA151, we tested the concentration-dependence of ODNR01 on inhibition of Th1 (IFN-γ), Th2 (IL-4) and Th17 (IL-17) cytokine production under three T-cell differentiation conditions. Incubation of ODNR01 with CD4^+^ T cells under Th0, Th1 and Th17 culture conditions resulted in a concentration-dependent decrease of IFN-γ and IL-17 production in the medium as determined by ELISA (Fig 1B). Under the same conditions, ODNR01 lacked effect on IL-4 production at any tested concentration, suggesting preferential inhibition of ODNR01 on differentiation of Th1 and Th17, but not of Th2. Real-time polymerase chain reaction (RT-PCR) analysis of cytokine mRNA levels yielded the same conclusion. ODNR01 (5–10 µM) inhibited IFN-γ and IL-17 expression significantly, but not that of IL-4 (Fig 1C). Flow cytometry histogram analysis further showed that ODNR01 inhibits Th1- and Th17-cell differentiation, but not Th2- or Treg-differentiation. When purified CD4^+^ T cells were cultured under Th0, Th1 and Th17 conditions, followed by intracellular staining with anti-T-bet and anti-RORγt antibodies to detect Th1- and Th17-cell populations (Cui et al, 2009; Yang et al, 2008), we found that under Th0 conditions, 5∼10 µM of ODNR01 reduced the percentage of Th1 and Th17 cells. Under Th1 and Th17 conditions, 5∼10 µM of ODNR01 inhibited the percentages of Th1 cells and Th17 cells significantly (Fig 1D and E). ODNR01 at any tested concentration, however, did not affect Th2 cells or CD4^+^CD25^+^Foxp3^+^ Treg frequency (Supporting Information Fig 1). At the same concentration (5 µM), ODNR01 was significantly stronger than ODNA151 in inhibiting the expression of IFN-γ and IL-17 or Th1 and Th17-cell frequencies, but had no inhibitory effect on IL-4 expression or Th2-cell frequencies in peripheral CD4^+^ T cells from Th1-biased C57BL/6 mice, while the control ODN1612 had no inhibitory activity (Supporting Information Fig 2A and B).

Splenic CD4^+^ T cells from Th2-biased Balb/c mice behaved similarly to those from Th1-biased C57BL/6 mice, which have constitutively suppressed Th2 responses (Bix et al, 1998). ELISA and RT-PCR determined that ODNR01 suppressed IFN-γ and IL-17 production dose-dependently at both protein and mRNA levels in CD4^+^ T cells from Balb/c mice under all three T-cell differentiation conditions (Th0, Th1 and Th17). At the same concentrations (1∼10 µM), however, ODNR01 did not affect IL-4 production at the protein and mRNA level (Supporting Information Fig 3A and B).

### ODNR01 binds to STAT1/3/4 and blocks phosphorylation

**Figure 2:**
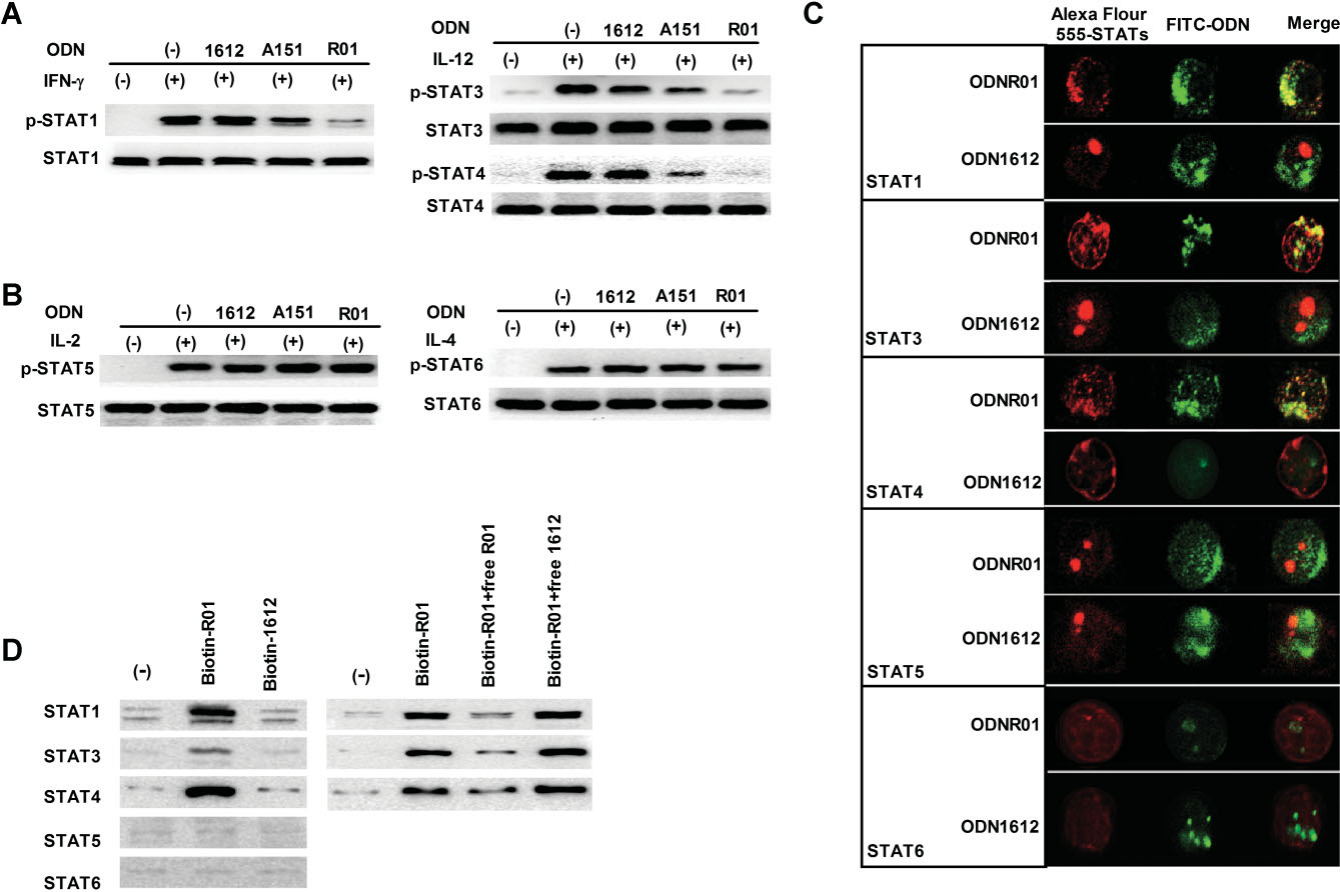
Immunosuppression of STAT1/3/4 phosphorylation by ODNR01 selective binding **A,B.** STAT and phospho-STAT Western blot analysis of anti-CD3/28 mAb-stimulated CD4^+^ T cells treated with indicated cytokines, with and without 5 µM of ODNR01, ODNA151 or ODN1612.**C.** Confocal microscopy to detect co-localization of FITC-conjugated ODNs (2.5 µM; green) with different Alex Fluor 555-conjugated anti-STAT rabbit polyclonal antibodies (red) in CD4^+^ T cells after 24 h of incubation.**D.** Left panels: Immunoblot analysis of STATs from CD4^+^ T cells pre-incubated with 5 µM biotinylated-ODNs, lysed, and precipitated with avidin agarose beads for STAT detection. Right panels: Immunoblot analysis of STATs from CD4^+^ T-cell lysates that were pre-incubated with 1 µM biotinylated ODN and 5 µM unlabelled ODN for 1 h and immunoprecipitated with avidin beads for detection of different STAT. The experiments were repeated three times with similar results.

To understand the mechanisms by which ODNR01 suppresses Th1 and Th17 differentiation, but not Th2 or Treg, we examined the STAT signalling pathways that direct T-cell subset differentiation and expansion (Zhu et al, 2010). IFN-γ (0.5 ng/ml) and IL-12 (0.5 ng/ml) stimulate the phosphorylation of STAT1 and STAT3/4, respectively (Afkarian et al, 2002; Harris et al, 2007; Thierfelder et al, 1996). While non-specific ODN1612 showed no effect on the phosphorylation of these STATs, 5 µM of ODNA151 and ODNR01 greatly inhibited the phosphorylation of STAT1 and STAT3/4 (Fig 2A). Under the same experimental conditions, ODNR01 appeared much more potent than ODNA151 in suppressing STAT1/3/4 phosphorylation (Fig 2A). IL-2 (10 ng/ml) and IL-4 (10 ng/ml) are commonly used to activate STAT5 and STAT6, which are critical to the differentiation of Treg and Th2, respectively (Burchill et al, 2007; Kaplan et al, 1996). Neither ODN1612 nor ODNA151 or ODNR01 inhibited STAT5 or STAT6 phosphorylation (Fig 2B), suggesting that ODNR01—but not ODN1612—inhibited the differentiation of Th1 and Th17 cells even more strongly than the known ODNA151, but not the differentiation of Treg or Th2 cells.

Inhibition of the phosphorylation of STAT1/3/4, but not of STAT5/6, by ODNR01, but not by ODN1612, suggested direct interaction of ODNR01 with cytoplasmic STAT1/3/4, but not with other STATs. To test this possibility, we performed confocal microscopy to monitor ODNR01–STAT interactions in cultured peripheral CD4^+^ T cells. In CD4^+^ T cells treated with ODNR01, ODNR01 (green) co-localized with STAT1/3/4 (red), but not with STAT5/6 (red; Fig 2C). In contrast, none of these STATs co-localized with the non-specific ODN1612 (green). Direct interaction between ODNR01 and STAT1/3/4 was explored using biotinylated ODNs in live CD4^+^ T cells and in CD4^+^ T-cell lysates. Avidin-coated agarose beads bound complexes of biotin-ODNR01 with STAT1/3/4, but not with STAT5/6 from live CD4^+^ T cells. Incubating biotin-ODNR01 with CD4^+^ T cells, followed by binding whole-cell lysate onto avidin-coated agarose beads, showed increased binding of STAT1/3/4, but not of STAT5/6 in the eluate from the avidin beads, as detected by subsequent immunoblot analysis using corresponding anti-STAT antibodies (Fig 2D, left panels). In contrast, the same method did not detect any differences between biotin-ODN1612-treated CD4^+^ T cells and untreated CD4^+^ T cells (Fig 2D, left panels), suggesting direct interaction between ODNR01 and STAT/1/3/4, but not among other tested signalling molecules or non-specific ODN1612. In CD4^+^ T-cell lysates, biotin-ODNR01 (1 µM) also bound to STAT1/3/4, an interaction that was blocked by excess free ODNR01 (5 µM), but not by free ODN1612 (5 µM; Fig 2D, right panel) further demonstrating the interaction of ODNR01 and STAT1/3/4 in CD4^+^ T cells.

### ODNR01 controls type-2 diabetes in obese mice

**Figure 3:**
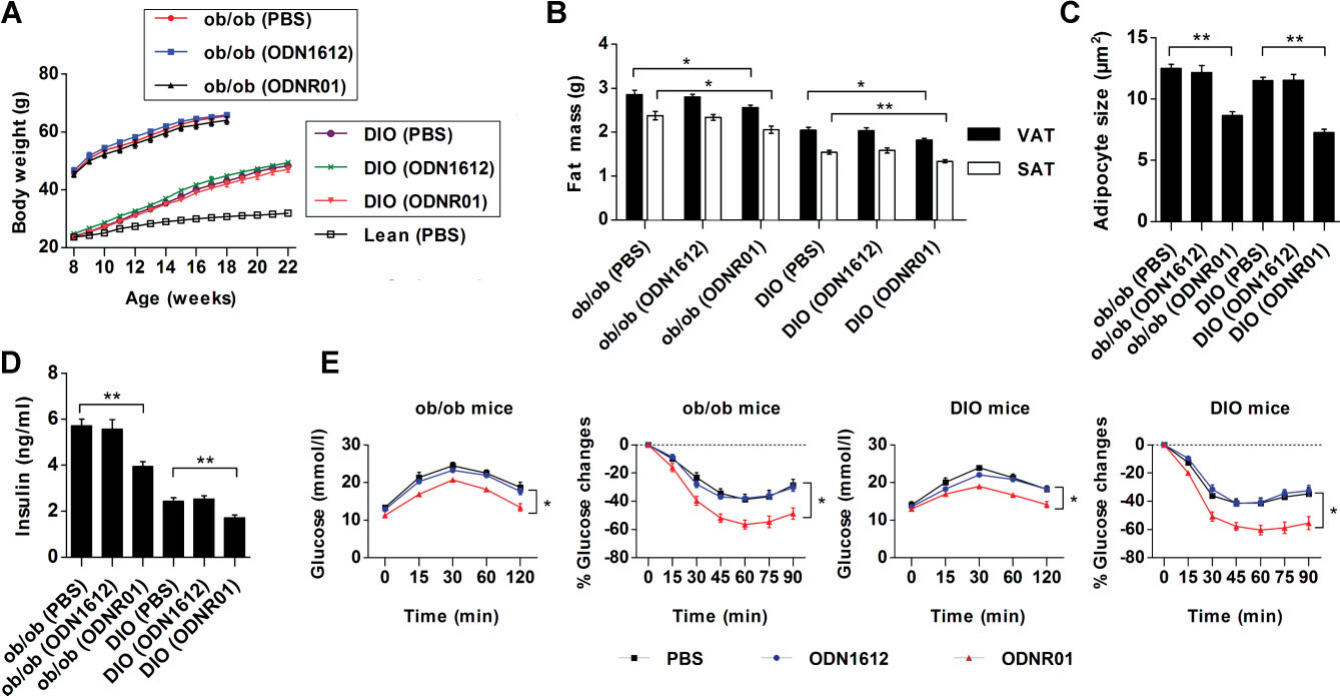
Effects of ODNs on obesity and diabetes in mice **A.** Body weight gain in DIO mice (on a high-fat diet, HFD) and *ob/ob* mice (on a normal chow diet, NCD) treated with ODNR01, negative control ODN1612 or PBS for indicated weeks (one dose/2 weeks).**B-E.** VAT and SAT fat mass (**B**), VAT adipocyte sizes (**C**), serum insulin levels (**D**), and glucose and insulin tolerance (**E**) from DIO mice and *ob/ob* mice at the end of treatment. *p < 0.05, **p *<* 0.01, *n* = 7∼8 mice per group.

Altered balance among Th1, Th2 and Treg contributes directly to obesity and diabetes. In WAT from DIO mice and *ob/ob* mice, increased IFN-γ–secreting Th1 and CD8^+^ T cells are accompanied by reduced numbers of Th2 cells [CD4^+^GATA-binding protein-3 (GATA-3)^+^] and regulatory forkhead box P3 (Foxp3)^+^ Treg cells (Feuerer et al, 2009; Nishimura et al, 2009; Winer et al, 2009a). While CD8^+^ T cells control the infiltration of macrophages and promote type-2 diabetes (Zhu et al, 2010), CD4^+^ T cells (presumably Th2 cells; Winer et al, 2009a, b) and Treg (Feuerer et al, 2009) reverse obesity and/or type-2 diabetes. ODNR01-mediated blockage of Th1 and Th17 differentiation may interrupt the balance between pro-inflammatory Th1/Th17 cells and anti-inflammatory Th2/Treg cells, which may protect DIO mice and *ob/ob* mice from T-cell inflammation-associated obesity and type-2 diabetes. Yet, we found no significant differences in body weight gain among the DIO groups or the *ob/ob* groups, regardless of whether mice received regulatory ODNR01, non-specific ODN1612 or phosphate buffered saline (PBS; Fig 3A). Consistent with these observations, we detected no significant differences in serum total cholesterol, high-density cholesterol (HDL) and triglyceride levels among these groups (Supporting Information Fig 4). Both visceral adipose tissue (VAT) and subcutaneous adipose tissue (SAT) fat mass, however, weighed about 0.3 g less in ODNR01-treated mice than in PBS-treated mice in both the *ob/ob* and DIO groups (Fig 3B). ODNR01-induced reductions in fat mass did not lead to significant changes in body weight gain from those of PBS- or ODN1612-treated mice (Fig 3A). Further, ODNR01-treated *ob/ob* mice and DIO mice had smaller adipocytes than controls (Fig 3C), suggesting that ODNR01 reduced WAT inflammation. Adipocyte size often associates with plasma adiponectin production (Bahceci et al, 2007; Skurk et al, 2007), but in our study, ODNRO1-induced adipocyte size changes did not affect plasma adiponectin levels (Supporting Information Fig 5). We also found that intraperitoneal administration (once every 2 weeks) of ODNR01, but not of ODN1612, significantly reduced fasting insulin levels in serum (Fig 3D) and improved glucose tolerance as well as insulin sensitivity (Fig 3E) in both DIO mice and *ob/ob* mice. Although we did not monitor mouse food and water intake due to insignificant body weight differences between the groups (Fig 3A), histologic examination of liver or gastrointestinal tract did not show differences between the groups. ODNR01, therefore, induced no discernable toxic effects and mitigated obesity-associated type-2 diabetes, but not body weight gain.

### ODNR01 regulates CD4^+^ T-cell subsets in obese mice

**Figure 4:**
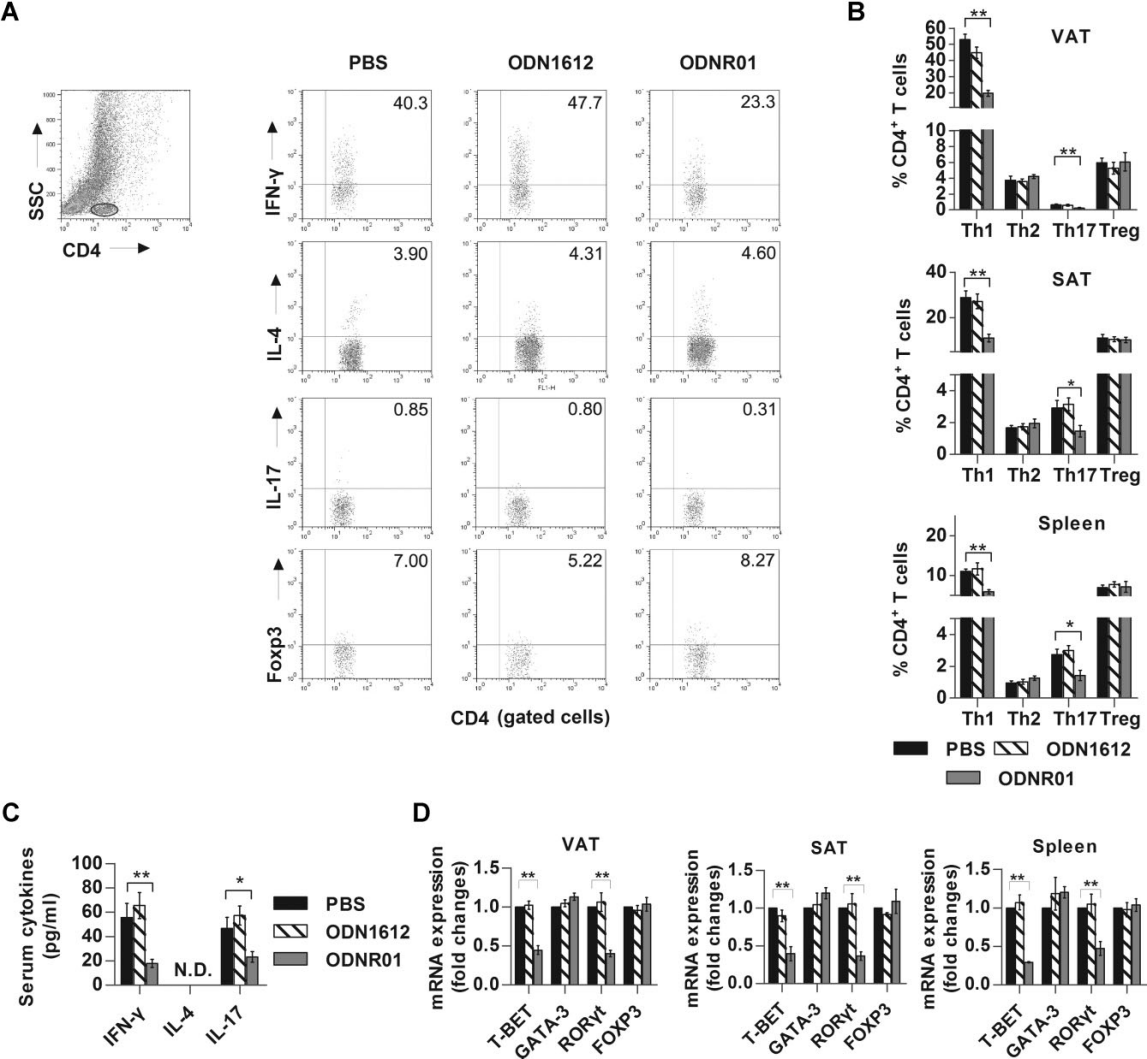
ODNR01 regulates CD4^+^ T-cell subsets in DIO mice Representative FACS plots of Th1, Th2, Th17 and Treg cells in VAT from DIO mice treated with different ODNs. CD4^+^ T cells were first gated on the SSC/CD4 plots, and then the expression of IFN-γ, IL-4, IL-17 and Foxp3 was analyzed in the gated CD4^+^ T cells.The frequencies of Th1, Th2, Th17 and Treg cells in VAT, SAT and spleen from DIO mice treated with different ODNs.ELISA determined serum IFN-γ, IL-4 and IL-17 concentrations in DIO mice treated with different ODNs.RT-PCR determined mRNA levels of transcription regulators (T-bet, GATA-3, RORγt and Foxp3) in sorted CD4^+^ T cells from VAT, SAT and spleen of DIO mice treated with different ODNs. *p < 0.05, **p *<* 0.01, ND: not detectable, *n* = 6∼8 mice per group.

CD4^+^ T cells, including Th2 and Treg cells, effectively control body weight gain and/or obesity-associated insulin sensitivity in mice (Feuerer et al, 2009; Winer et al, 2009a, b). ODNR01 effectively inhibited Th1- and Th17-cell differentiation *in vitro* but did not affect Th2 or Treg (Figs 1 and 2), leading to a shift in balance toward Th2 and Treg and improved insulin sensitivity in obese mice (Fig 3D and E). We examined T-cell subsets in VAT, SAT and spleen from DIO mice (Fig 4). Flow cytometry analysis of the SVF preparation from VAT of DIO mice demonstrated that ODNR01 reduced CD4^+^IFN-γ^+^ (Th1) and CD4^+^IL-17^+^ (Th17) cells, but did not affect CD4^+^IL-4^+^ (Th2) and CD4^+^Foxp3^+^ (Treg) cells (Fig 4A). In contrast, non-specific ODN1612 did not affect any of these CD4^+^ T-cell subsets. We made similar observations in SAT and spleen from the same DIO mice (Fig 4B). Serum Th subset characteristic cytokines showed similar regulation by ODNR01. Both serum Th1 cytokine IFN-γ and Th17 cytokine IL-17 levels were significantly lower in mice treated with ODNR01, but not with ODN1612, although serum IL-4 levels were undetectable (Fig 4C). These results suggest that ODNR01 regulates Th-cell subsets *in vivo*, which may explain reduced glucose tolerance and enhanced insulin sensitivity in DIO mice and *ob/ob* mice (Fig 3D and E). To test this conclusion further, we isolated CD4^+^ T cells from DIO mice that were treated with ODNR01, ODN1612 or PBS and examined the mRNA levels of different Th subset-specific key transcription regulatory molecules. ODNR01 inhibited significantly the mRNA levels of transcription factor T-bet and orphan nuclear receptor RORγt—which are Th1- and Th17-cell signature transcriptional regulators—in VAT, SAT and spleen. Under the same conditions, ODNR01 did not affect the expression of transcription factor GATA-3 or transcription regulator Foxp3, both of which belong to the Th2 and Treg cell-transcription machinery (Fig 4D). Under all tested conditions, the non-specific ODN1612 did not affect any of these transcriptional regulators. Study of CD4^+^ T cells from VAT, SAT and spleen from *ob/ob* mice yielded similar observations (Supporting Information Fig 6).

### ODNR01 reduces adipose tissue macrophage accumulation and M1-associated inflammatory factor expression

**Figure 5:**
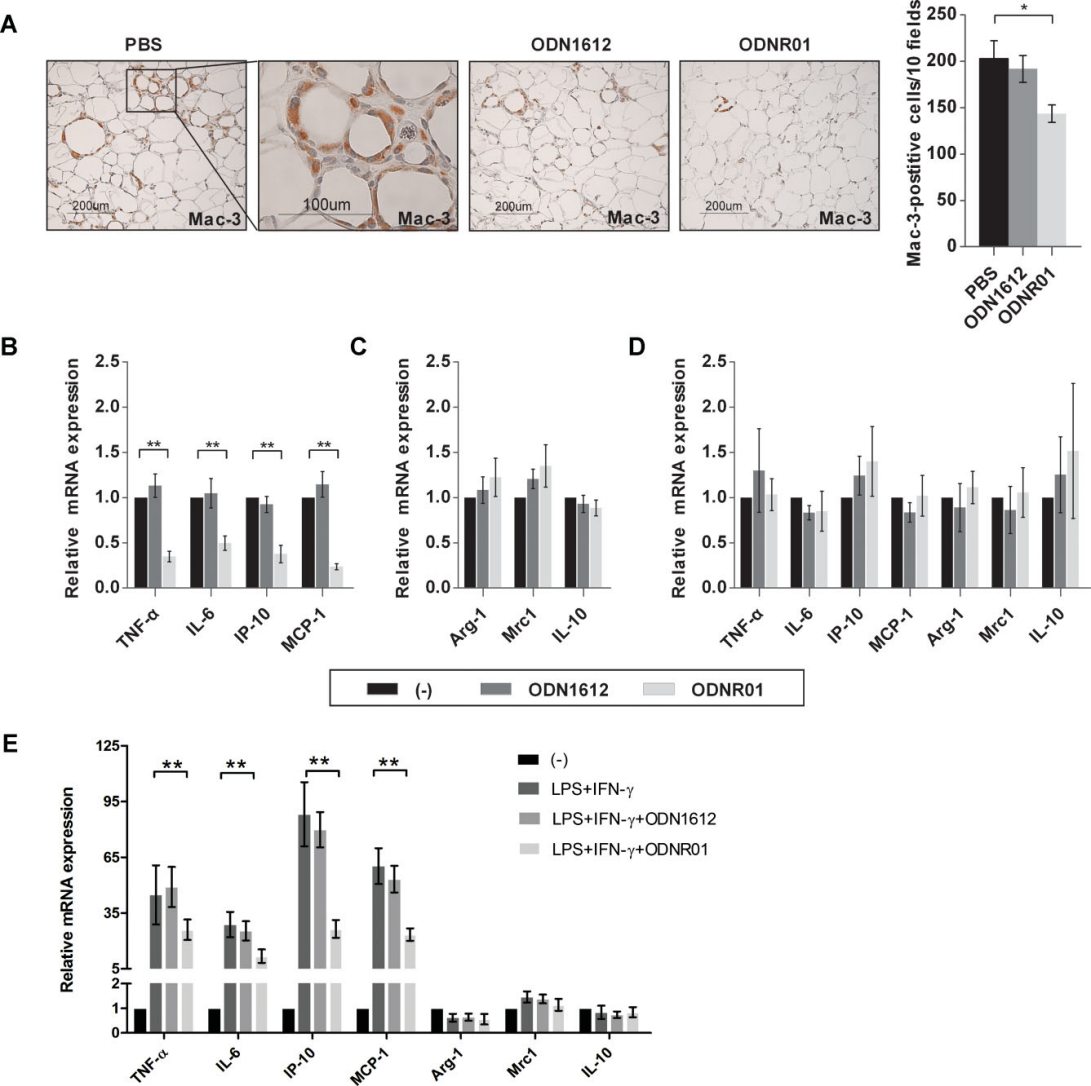
Macrophage accumulation and M1 inflammatory factor expression in VAT from DIO mice treated with or without different ODNs or *in vitro* cultured macrophages **A.** Immunostaining of VAT with rat anti-mouse Mac-3 mAb. Graph represents the number of Mac-3^+^ cells in each 10 fields.**B-E.** RT-PCR determined mRNA levels of (**B**) M1 markers (TNF-α, IL-6, IP-10 and MCP-1) and (**C**) M2 markers (Arg1, Mrc1 and IL-10) in VAT after normalizing to GAPDH. Fold change was calculated relative to PBS group. RT-PCR determined mRNA levels (normalized to GAPDH) of M1 markers (TNF-α, IL-6, IP-10 and MCP-1) and M2 markers (Arg1, Mrc1 and IL-10) in untreated BMDMs (**D**) or BMDMs that were stimulated with LPS (10 ng/ml) and IFN-γ (100 U/ml) for 24 h (**E**), with or without 5 µM ODNR01 or ODN1612. Fold change was calculated relative to medium group (−). Data represent three independent experiments. *p < 0.05; **p *<* 0.01, *n* = 4∼6.

WAT pro-inflammatory cells, such as CD8^+^ T cells and mast cells, directly influence macrophage accumulation, thereby affecting adipose tissue growth, glucose metabolism and insulin sensitivity (Liu et al, 2009; Nishimura et al, 2009). Reduced pro-inflammatory Th1 and Th17 cells in ODNR01-treated DIO mice or *ob/ob* mice may also result in low macrophage content in WAT. VAT immunostaining with anti-Mac-3 mAb revealed significantly fewer Mac-3^+^ cells in VAT from ODNR01-treated DIO mice than in those from PBS-treated or ODN1612-treated mice (Fig 5A). VAT from ODNR01-treated DIO mice contained significantly fewer classically activated pro-inflammatory M1 macrophage cytokines and chemokines [tumour necrosis factor alpha (TNF-α), IL-1, IP-10 and monocyte chemoattractant protein (MCP-1)] than did VAT from PBS-treated or ODN1612-treated mice (Fig 5B). In contrast, neither ODNR01 nor ODN1612 affected alternatively activated anti-inflammatory M2 macrophage cytokines (Arg-1, Mrc-1 and IL-10; Fig 5C). These observations suggest that ODNR01 regulates M1 macrophage polarization in VAT from DIO mice. Study of SAT from DIO mice and in VAT and SAT from *ob/ob* mice yielded similar observations (Supporting Information Fig 7). Of note, when cultured naïve bone marrow-derived macrophages (BMDMs) were used, neither ODNR01 nor ODN1612 affected M1 or M2 cytokine and chemokine expression (Fig 5D), suggesting that ODNR01 affected macrophage polarization under certain conditions, such as in the presence of inflammation or under activation stimulation from other inflammatory cells or cytokines. To test this hypothesis, we performed the same experiments as in Fig 5D. Instead of using naïve BMDMs, we activated BMDMs with lipopolysaccharide (LPS, 100 ng/ml) and Th1 cytokine IFN-γ (100 U/ml; Khallou-Laschet et al, 2010). Consistent with our hypothesis, under these conditions, ODNR01 showed significant inhibitory effects on M1 marker expression, but remained ineffective on M2 marker expression (Fig 5E). As in CD4^+^ T cells (Fig 2C), ODNR01 but not ODN1612 also interacted with STAT1/3/4, but not with STAT5/6 in BMDMs. Confocal microscopy demonstrated that ODNR01 colocalized with STAT1/3/4, but not with STAT5/6 in BMDMs (Supporting Information Fig 8). Therefore, ODNR01 targets not only T cells, but also macrophages under inflammatory conditions.

### Effects of ODNR01 on glucose homeostasis are CD4^+^ T-cell-dependent

**Figure 6:**
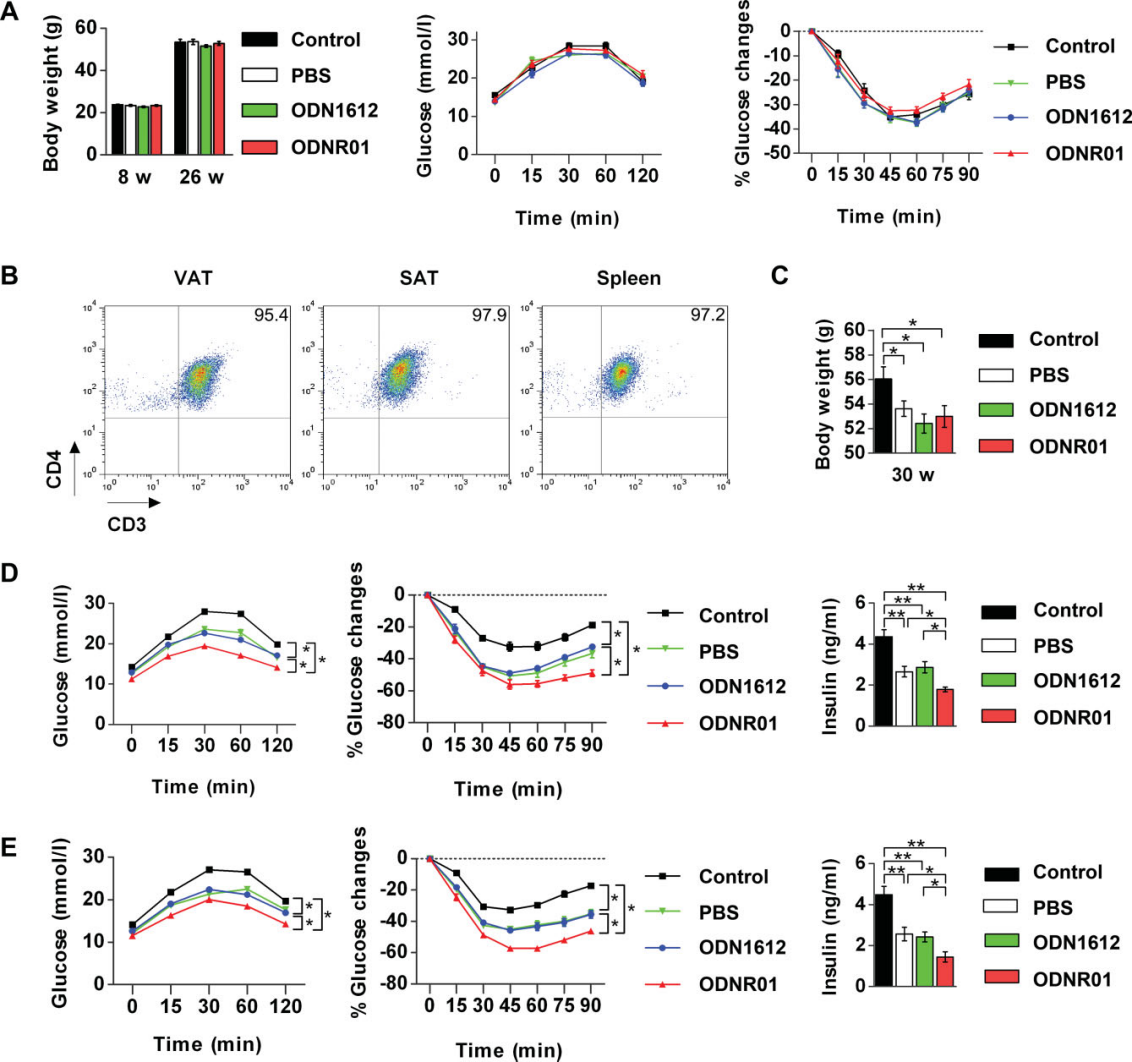
CD4^+^ T-cell-dependent effects of ODNR01 on glucose homeostasis *Rag1*^*−/−*^ mouse body weight at 8 weeks or after consuming 18 weeks of a HFD along with ODNR01 or ODN1612 treatment (one dose/2 weeks), glucose tolerance test (GTT) and insulin tolerance test (ITT) at the end of HFD and ODN treatment.FACS plots to detect CD3^+^CD4^+^ T cells in VAT, SAT and spleen from *Rag1*^*−/−*^ mice that consumed HFD for 18 weeks (no ODN treatments), then received WT naive CD4^+^ T-cell intraperitoneal transfer (5 × 10^6^ mouse^−1^) at week 26 and consumed HFD for an additional 4 weeks while receiving ODN treatments.Body weight of *Rag1*^*−/−*^ recipients from **B**.GTT, ITT and serum insulin levels in *Rag1*^*−/−*^ recipients from **B**.GTT, ITT and serum insulin levels from *Rag1*^*−/−*^ mice that consumed a HFD for 18 weeks (no ODN treatments), received adoptive transfer of *in vitro* ODN-treated WT CD4^+^ T cells, and consumed a HFD for another 4 weeks. Control: no reconstitution or treatment but HFD for the same time period; PBS: CD4^+^ T-cell-reconstituted mice treated with PBS or mice reconstituted with PBS-treated CD4^+^ T cells. *p *<* 0.05, **p *<* 0.01, *n* = 6∼8.

To examine whether the effects of ODNR01 on controlling glucose and insulin homeostasis in DIO mice and *ob/ob* mice required CD4^+^ T cells, we studied T-cell-deficient Rag1-null (*Rag1*^*−/−*^) mice, which also gain body weight after consuming a high-fat diet (HFD; Winer et al, 2009a, b). *Rag1*^*−/−*^ mice at 8 weeks of age started consuming a HFD while receiving intraperitoneal PBS, ODNR01 or ODN1612 once every other week. After 18 weeks of a HFD, *Rag1*^*−/−*^ mice receiving PBS or no treatment (control) gained body weight consistently and developed type-2 diabetes. ODNR01 and ODN1612 did not affect body weight gain (Fig 6A, left panel), glucose tolerance (Fig 6A, middle panel) or insulin sensitivity (Fig 6A, right panel), unlike in *ob/ob* mice or DIO WT mice (Fig 3D and E). ODNR01 therefore did not improve diabetes in the absence of T cells, suggesting that ODNR01 activity in improving diabetes is T-cell-dependent. To test this hypothesis, we adoptively transferred *in vitro* prepared CD4^+^ T cells from WT mice into *Rag1*^*−/−*^ mice that had consumed 18 weeks of a HFD. At 4 weeks after CD4^+^ T-cell adoptive transfer, we detected donor CD3^+^CD4^+^ T cells in VAT, SAT and spleen (Fig 6B). As previously reported (Winer et al, 2009a, b), reconstitution of CD4^+^ T cells (PBS group) significantly reduced body weight gain (Fig 6C), glucose tolerance, insulin sensitivity and serum insulin levels (Fig 6D). Although ODN1612 did not further reduce obesity or improve diabetes compared to those treated with PBS, ODNR01 further improved type-2 diabetes significantly, including glucose tolerance, insulin sensitivity and serum insulin levels (Fig 6D). As in the experiment presented in Fig 3A, ODNR01 did not affect body weight gain (Fig 6C). Consistent with the findings from DIO mice presented in Fig 5B/5C, VAT from *Rag1*^*−/−*^ mice showed significantly reduced expression levels of M1 cytokines and chemokines (TNF-α, IL-1, IP-10 and MCP-1) after receiving donor CD4^+^ T cells. ODNR01, but not control ODN1612, further reduced these M1 markers. In contrast, CD4^+^ T-cell reconstitution increased significantly all tested M2 markers (Arg-1, Mrc-1 and IL-10) in VAT from *Rag1*^*−/−*^ mice. Neither ODNR01 nor control ODN1612 further affected the expression of these M2 markers (Supporting Information Fig 9). ODNR01 therefore improved type 2 diabetes by targeting CD4^+^ T cells and consequently M1-cell polarization in DIO mice or *ob/ob* mice. To test this hypothesis further, we reconstituted *Rag1*^*−/−*^ mice that had consumed 18 weeks of a HFD with CD4^+^ T cells that had been pre-incubated with ODNR01, ODN1612 or vehicle (PBS) for 3 days. After consuming 4 weeks of a HFD, *Rag1*^*−/−*^ mice receiving ODNR01-treated CD4^+^ T cells demonstrated further improvement of glucose tolerance, insulin sensitivity and serum insulin levels compared to non-reconstituted mice or those receiving PBS-treated or ODN1612-treated CD4^+^ T cells (Fig 6E).

## DISCUSSION

Obesity associates with inflammation in adipose tissue. Influx of macrophages, T cells, mast cells and other untested inflammatory cells in adipose tissues releases pro-inflammatory mediators upon development of chronic caloric excess-driven obesity. These inflammatory cells cause insulin resistance and its associated complications (Feuerer et al, 2009; Liu et al, 2009; Nishimura et al, 2009; Winer et al, 2009a; Weisberg et al, 2003). Immunosuppressive ODNs containing TTAGGG motifs inhibit the phosphorylation of STAT1/4 and block the differentiation of Th1 cells and production of their inflammatory mediators (Gursel et al, 2003; Shirota et al, 2004, 2005). This study used optimized ODNs that replaced the TTAGGG motifs in ODNA151 with TAGGG yielding a potent regulatory ODNR01 that suppressed Th1/Th17 differentiation and subsequently M1 macrophage polarization, without affecting Th2 and Treg differentiation, and lacking evident toxicity. Unbalanced regulation of Th1/Th17 *versus* Th2/Treg as well as impaired M1 macrophage polarization and recruitment to WAT by ODNR01 induced protective effects on glucose and insulin homeostasis in DIO mice and *ob/ob* mice.

In addition to CD4^+^ lymphocytes, many other cell types—including macrophages (Weisberg et al, 2003), mast cells (Liu et al, 2009), γδT cells (Zúñiga et al, 2010) and eosinophils (Wu et al, 2011)—also regulate glucose homeostasis. ODNR01 may alleviate insulin resistance through regulating differentiation or functions of other cell types. Therefore, we used ODNR01 to treat lymphocyte-free Rag1-null DIO mice or Rag1-null DIO mice receiving CD4^+^ T-cell adoptive transfer. Our experiments demonstrated that ODNR01 had no effect on obesity and insulin resistance in lymphocyte-free Rag1-null DIO mice, while it improved insulin sensitivity in Rag1-null DIO mice that received CD4^+^ T-cell adoptive transfer suggesting that the benefits of ODNR01 in obese mice and in WAT are CD4^+^ T-cell-dependent.

Obesity and associated type-2 diabetes display a Th1-bias, based on an existing imbalance in obese WAT between dominant Th1 responses and reduced Th2 response (Feuerer et al, 2009; Nishimura et al, 2009; Rocha et al, 2008; Winer et al, 2009a). A signature Th1 cytokine, IFN-γ elevates expression of MCP-1, TNF-α and RANTES and macrophage accumulation in obese WAT. Obese IFN-γ-deficient animals had decreased inflammatory cell accumulation in WAT and better glucose tolerance than WT control animals (Rocha et al, 2008) as seen in ODNR01-treated DIO mice or *ob/ob* mice (Figs 3D, E, and 5A). Recent work suggests that Th2 cells exert protective functions in insulin resistance during obesity (Winer et al, 2009a). Adoptive transfer of CD4^+^ T cells to T-cell-deficient *Rag1*^*−/−*^ mice reduced obesity and improved diabetes (Winer et al, 2009a). The current study used ODNR01 to regulate the CD4^+^ T-cell balance by blocking Th1- and Th17-cell differentiation. ODNR01 significantly reduced macrophage accumulation and M1 inflammatory factor expression in WAT and improved glucose and insulin sensitivity in obese mice. These results suggested that ODNR01 induced protective effects on glucose and insulin homeostasis *in vivo* by decreasing Th1-cell functions and enhancing Th2-cell activities, providing additional evidence that Th1/Th2-cell counterbalance controls insulin resistance in obese mice. Two important questions, however, emerged from our observations.

Earlier observations (Winer et al, 2009a) and this study (Fig 6C) demonstrated that reconstitution of total CD4^+^ T cells reduces body weight gain in *Rag1*^*−/−*^ mice. But inhibition of Th1 and Th17 and concomitant increase of Th2 and Treg by ODNR01 in C57BL/6 WT DIO mice, *ob/ob* mice or CD4^+^ T-cell-reconstituted *Rag1*^*−/−*^ mice did not affect body weight. Reduced obesity in CD4^+^ T-cell-reconstituted *Rag1*^*−/−*^ mice therefore might not associate with Th2 or Treg, but rather with other uncharacterized CD4^+^ T-cell populations. Previous Treg gain-of-function and loss-of-function studies (Feuerer et al, 2009) demonstrated a role of Treg cells in insulin resistance, but the potential effect of Treg cells on body weight gain had not been fully studied. Although Winer et al (2009a) demonstrated that Th2 cells might control body weight gain after CD4^+^ T-cell transfer, recent evidence suggests that Th1 and Th2 cells do not control body weight gain. In DIO mice, the absence of Th1 cytokine IFN-γ led to reductions in WAT macrophages, adipocyte size and expression of inflammatory molecules (TNF-α and MCP-1). These mice demonstrated increased glucose sensitivity but showed no difference in body weight gain compared with WT control mice (Rocha et al, 2008), suggesting a negligible role of Th1 cells in controlling body weight. IL-33, a member of the IL-1 family, induces helper T cells, mast cells and other inflammatory cells to produce Th2 cytokines (Schmitz et al, 2005). WAT releases Th2 cytokines (IL-5, IL-13 and IL-10) after stimulation with IL-33 *in vitro* (Miller et al, 2010). In *ob/ob* mice, administration of recombinant IL-33 polarized the ATMs toward an M2 alternatively activated phenotype in WAT, reduced WAT adiposity (reduced adipocyte size and epididymal fat mass) and improved glucose and insulin tolerance. Activation of Th2 cells, however, did not change body weight gain (Miller et al, 2010). The IFN-γ and IL-33 studies support the same conclusion as the current study: that Th1 or Th2 cells (and possibly Th17 or Treg cells) may affect WAT inflammation, adiposity and glucose and insulin tolerance, but do not affect body weight gain. Reduced body weight in CD4^+^ T-cell-reconstituted *Rag1*^*−/−*^ mice (Winer et al, 2009a; Fig 6C) may derive from a different CD4^+^ cell population—a hypothesis that merits further investigation.

Second, in the current study, inactivation of both Th1 and Th17 cells improved insulin resistance (Figs 3D, E and 6D and E). Given the observations from anti-CD3 antibody-treated DIO mice that depletion of Th1 cells or deficiency of Th1 cytokine IFN-γ reversed adipose tissue inflammation and insulin resistance (Winer et al, 2009a; Rocha et al, 2008), inactivation of Th1 cells will suffice to improve insulin resistance in ODNR01-treated mice. Therefore, Th17-cell differentiation may not necessarily contribute to improved insulin resistance (Figs 3D and E and 6D and E). We currently do not know the exact role of Th17 cells in obesity or insulin resistance. Like Th1 cells, Th17 cells contribute to inflammatory autoimmunity (Bettelli et al, 2008), but their possible roles in insulin resistance remains unclear. Clinical studies revealed increased serum IL-17 levels in obese patients (Sumarac-Dumanovic et al, 2009), while obesity selectively promotes Th17-cell lineage expansion, thereby exacerbating pathogenesis in IL-17-dependent mouse models of EAE and trinitrobenzene sulphonic acid-induced colitis (Pini & Fantuzzi, 2010; Winer et al, 2009b). Recent studies also demonstrate, however, that IL-17 reduces adipogenesis and can delay the development of obesity (Zúñiga et al, 2010). Absence of IL-17 increases body weight gain in mice. While young adult IL-17-deficient mice were more sensitive than WT control mice to glucose and insulin challenges, increased glucose tolerance and insulin sensitivity in IL-17-deficient mice diminished after consumption of a HFD for 14–16 weeks (Zúñiga et al, 2010). These observations suggest that IL-17 participates in body weight gain, but only negligibly contributes to insulin resistance in mice after consuming a HFD. In the present study, although ODNR01 decreased Th17 frequencies and IL-17 concentrations in obese mice, body weight gain did not change among all groups. Increased body weight gain due to IL-17 inhibition may have been obscured by reduced overall WAT inflammation after ODNR01 treatment. Further, minimal effects of IL-17 in insulin resistance in DIO mice (Zúñiga et al, 2010) may support a major role of Th1-cell subsets in type-2 diabetes from the current study.

This study demonstrated that regulatory ODNR01 limits immune activation associated with obesity and diabetes. Such activity of regulatory ODNR01 might benefit humans or animals with other inflammatory diseases, such as cardiovascular disease, cancers or infectious diseases—a hypothesis that merits further investigation. But a broad immunosuppressive activity of ODNR01 or other regulatory ODNs on the overall immune system, rather than on a specific tissue (*e.g.* adipose tissue), may predispose to opportunistic infections or impair tumour defenses, although such adverse effects have not emerged to date. Additional research is needed to determine whether this compromised immunity of regulatory ODN outweighs the agent's therapeutic effect in humans.

Taken together, these results identify an ODN that modulates mouse WAT inflammation and type-2 diabetes by regulating Th1/Th17 subset differentiation, which in turn decreases macrophage accumulation and M1 inflammatory factor expression in WAT. These results affirm the regulatory role of adaptive immunity in the metabolic disorders associated with obesity and present a potential therapeutic avenue for patients (Klinman et al, 2009) with, or predisposed to, type-2 diabetes—although orally available ODNs have not been tested.

## MATERIALS AND METHODS

### ODNs

Unlabelled, biotin-labelled and FITC-labelled ODNs were synthesized at Invitrogen, Shanghai, China. All ODNs used in this study were phosphorothioated (Invitrogen) to prevent degradation (Brown et al, 1994). Sequences of the ODNs used in this study were as follows:

regulatory ODNR01: TAGGGTAGGGTAGGGTAGGG;
regulatory ODNR02: TTTAGGGTTAGGGTTAGGG;
regulatory ODNR03: TCAGGGTCAGGGTCAGGGTCAGGG;
regulatory ODNR04: TAAGGGTAAGGGTAAGGGTAAGGG;
suppressive ODNA151: TTAGGGTTAGGGTTAGGGTTAGGG;
and control ODN1612: GCTAGATGTTAGCGT.

There was no detectable endotoxin or protein contamination in these ODN preparations.

### CD4^+^ T-cell and macrophage preparation

Peripheral CD4^+^ T cells from splenocytes of 8-week-old Th1-biased C57BL/6 mice and Th2-biased Balb/c mice were MACS-sorted using a mouse CD4^+^ T-cell isolation kit II (Miltenyi Biotec Inc., Auburn, CA), according to the manufacturer's instructions. CD4^+^ T-cell purity was confirmed by flow cytometry.

BMDMs were isolated from the femurs and tibias of 8-week-old C57BL/6 mice and differentiated into macrophages by incubating bone marrow cells in Iscove's modified Dulbecco's medium (IMDM) supplied with 10% FCS, 1% glutamine and 10 ng/ml macrophage-colony stimulating factor (M-CSF, R&D Systems, Minneapolis, MN), for 7 days as described previously (Odegaard et al, 2007).

### Cell culture

To evaluate ODN activities on CD4^+^ T cells, CD4^+^ T cells (2.5 × 10^6^/ml) were cultured in a complete medium (RPMI 1640 medium and 10% foetal bovine serum) in the presence of plate-bound anti-CD3 (1 µg/ml) and soluble anti-CD28 (1 µg/ml) monoclonal antibodies (mAb, BD Pharmingen, San Diego, CA) to activate T-cell receptors (TCRs). Th1 mixture [IL-12 (10 ng/ml) and anti-IL-4 antibody (10 µg/ml)] or Th17 mixture [IL-6 (20 ng/ml), TGF-β (3 ng/ml), IL-23 (10 ng/ml), anti-IFN-γ and anti-IL-4 antibodies (10 µg/ml)] were added to drive Th1 and Th17 polarization, respectively. Cells were cultured with or without different concentrations of ODNs for 3 days. After brief washes, 2 × 10^6^ ml^−1^ viable cells were re-stimulated with 1 µg/ml plate-bound anti-CD3 mAb for another 48 h. Culture supernatants were collected and stored at −80°C for cytokine ELISA analysis. In some experiments, cells after 3–4 days of treatment with ODNs were used for RT-PCR, flow cytometric analysis or mouse adoptive transfer.

To evaluate the effect of ODNR01 on BMDM differentiation, we stimulated BMDMs with ODNR01 (5 µM) or ODN1612 (5 µM) for 48 h (Baek et al, 2001), purified total RNA using Trizol (Invitrogen, Carlsbad, CA) and determined relative mRNA levels using RT-PCR. To study the effects of ODNs on CD4^+^ T-cell differentiation signal pathways, anti-CD3/28 mAb-stimulated CD4^+^ T cells were incubated with different stimuli (*e.g.* IFN-γ, IL-12, IL-2 and IL-4), washed with PBS, and lysed in ice-cold PhosStop lysis buffer containing protease and phosphatase inhibitors (Roche Applied Science, Indianapolis, IN). Protein extracts were used for Western blot analysis.

### Confocal microscopy

CD4^+^ T cells or BMDMs were incubated with FITC-labelled ODN (2.5 µM) for 24 h at 37°C in a complete RPMI 1640 medium containing 10% foetal bovine serum (CD4^+^ T cells) or DMEM containing 5% foetal bovine serum (BMDMs). Cells were washed and fixed with 4% paraformaldehyde, permeabilized with 1% Triton-100 in PBS, and stained with rabbit anti-STAT1/3/4/5/6 antibodies (1 µg/µl, Signalway Antibody, Pearland, TX), followed by Alex Fluor 555 or 670-labelled anti-rabbit antibody. The cells were analyzed with confocal microscopy for subcellular localization of FITC and Alex Fluor 555 or 670 (Nikon A1Si; Nikon instruments, Japan).

### Ligand binding studies

CD4^+^ T cells were incubated for 4 h with 5 µM biotinylated-ODN at 37°C before lysis, and subjected to microcentrifugation to remove nuclear debris. Alternatively, clarified cellular lysates were incubated with 1 µM biotinylated-ODN and 5 µM free ODN for 2 h at 4°C. Twenty-five microliters of avidin-coated agarose (Sigma–Aldrich, St. Louis, MO) were added to 200 µl of lysate and rotated for 30 min at 4°C. Pellets were washed four times in lysis buffer, boiled for 10 min, and then analyzed by Western blot (Latz et al, 2004).

### Mice

Wild-type C57BL/6 mice, genetically generated obese (*ob/ob*) mice, and Rag1-deficient (*Rag1*^*−/−*^) mice were purchased from the Jackson Laboratories (Bar Harbor, ME). Regulatory ODNR01 and non-specific control ODN1612 were dissolved in 300 µl PBS for intraperitoneal injection once every 2 weeks. Eight-week-old mice received either a normal chow diet (NCD) or a HFD (D12492, Research Diets, Inc., New Brunswick, NJ) for 10 weeks (*ob/ob* mice, NCD), 14 weeks (C57BL/6 mice, HFD) or 22 weeks (*Rag1*^*−/−*^ mice, HFD). ODNs at 12 mg/kg body weight were given to C57BL/6 mice, *Rag1*^*−/−*^ mice and *ob/ob* mice. For CD4^+^ T-cell reconstitution experiments, 8-week-old *Rag1*^*−/−*^ mice consumed a HFD for 18 weeks and then received CD4^+^ T cells (5 × 10^6^ mouse^−1^) or CD4^+^ T cells pre-incubated with ODNR01 (5 µM), ODN1612 (5 µM) or no ODN (5 × 10^6^ mouse^−1^). Mice continued on a HFD for four more weeks before characterization. The Harvard Medical School Standing Committee on Animals approved all animal experiments.

Body weight was monitored weekly. Glucose tolerance tests (GTTs) and insulin tolerance tests (ITTs) were conducted in mice before adoptive transfer and at sacrifice. For GTTs, we gave fasted (16 h) mice 1 g of glucose/kg body weight; for ITTs, we gave 0.75 U/kg body weight of human regular insulin (Novo Nordisk, Bagsværd, Danmark; 2 U/kg in *ob/ob* mice).

**The paper explained**

PROBLEM:

Regulatory ODNs may be used to suppress pro-inflammatory T-cell subsets, thereby improving body weight gain, glucose metabolism and insulin sensitivity in obese subjects.

RESULTS:

This study developed a novel, non-toxic and potent immunosuppressive regulatory ODN after screening of a set of new ODNs, based on structural modifications of existing ODNs. This new ODN can selectively block pro-inflammatory T cells (Th1 and Th17) by binding and inactivating the essential STAT signalling pathways of Th1 and Th17, without affecting those of anti-inflammatory T-cell subsets (Th2 and regulatory T cells). In both diet-induced and genetically modified obese mice, this new ODN enhanced glucose metabolism and insulin sensitivity and suppressed adipose tissue inflammation, including reduced adipose tissue fat cell size and impaired pro-inflammatory macrophage accumulation and polarization. These ODN phenotypes disappeared in T-cell-deficient mice, but reappeared after mice received T-cell-adoptive transfer—and therefore are T-cell-dependent.

IMPACT:

As immunostimulatory ODN has been used in several clinical trials in cancer patients, this immunosuppressive ODN may have therapeutic potential in patients with obesity, diabetes and associated complications.

### Stromal vascular fraction, splenocyte isolation and flow cytometry analysis

We isolated SVF using previously described methods, with some modifications (Rocha et al, 2008). Epididymal VAT and SAT were removed and minced into small pieces (∼2 mm) in cold PBS. Tissues were digested in RPMI-1640 containing 35 µg/ml Liberase TM (Roche Applied Science) and 50 U/ml DNase I (Sigma) at 37°C for 45 min. Digested tissues were passed through a 70-µm cell strainer (BD Biosciences) and flow-through centrifuged. After aspirating the supernatant, red blood cells were lysed with an ACK lysing buffer (Gibco). The remaining cells, as SVF, were washed with RPMI-1640 twice and used for flow cytometry or CD4^+^ T-cell sorting. Splenocytes were isolated as previously described (Cheng et al, 2008).

The following antibodies were used for flow cytometry: anti-CD3-APC, anti-CD4-FITC, anti-IFN-γ-PE, anti-IL-17-PE-cy7, anti-IL-4-APC and anti-Foxp3-APC (all from eBioscience, Inc., San Diego, CA). For surface staining, cells were incubated with antibodies for 20 min at 4°C. For Treg staining, cells were fixed and permeabilized according to the manufacturer's instructions (eBioscience, Inc.) before adding the anti-Foxp3 antibody. For the Th1, Th2 and Th17 staining, cells were re-stimulated with 50 ng/ml phorbol 12-myristate 13-acetate (PMA) and 500 ng/ml ionomycin in the presence of GolgiPlug for 6 h, fixed, and permeabilized before adding anti-IFN-γ, IL-4 or IL-17 antibodies. Isotype controls were used to correct compensation and to confirm antibody specificity. All samples were analyzed using flow cytometry on a FACSCalibur (BD Biosciences).

### Immunohistochemistry

Visceral adipose tissue was fixed and embedded in paraffin. Section staining was performed with a rat anti-mouse Mac-3 mAb (1:900, BD Pharmingen) as described previously (Liu et al, 2009). Positive cells were counted in 10 consecutive visual fields at the same magnification.

### Real-time PCR

Total RNAs from CD4^+^ T cells, BMDMs and adipose tissues were prepared using a TRIzol reagent (Invitrogen). IFN-γ, IL-17, IL-4, T-bet, RORγt, GATA-3, Foxp3, TNF-α, IL-6, interferon-inducible protein (IP)-10, MCP-1, arginase 1 (Arg1), mannose receptor C-type 1 (Mrc1), IL-10 and glyceraldehyde-3-phosphate dehydrogenase (GAPDH) cDNA templates were synthesized using a random hexamer primer and RNase H-reverse transcriptase (Invitrogen). RT-PCR was performed and analyzed on an ABI Prism 7900 Sequence Detection system (Applied Biosystems, Carlsbad, CA) using SYBR Green Master Mix (Takara, Madison, WI). The relative expression level of each gene was normalized with housekeeping gene GAPDH and calculated by the 2^−ΔΔCt^ methods.

### Western blotting

Protein extracts from CD4^+^ T cells were used for Western blots. Lysate protein concentrations were determined using a BCA protein kit (Pierce, Rockford, IL). Protein extract (25 µg) from each sample was separated on a 9% sodium dodecyl sulphate–polyacrylamide gel electrophoresis (SDS–PAGE) for Western blot analysis. Western blots were probed using primary antibodies to phosphorylated-STAT1, STAT3, STAT4, STAT5, STAT6 and corresponding non-phosphorylated STATs (all at 1:1000 dilution; Cell Signaling Technology, Inc., Danvers, MA). Horseradish peroxidase-conjugated anti-rabbit IgG (1:20,000, Sigma) was used as a secondary antibody. Proteins were detected using an ECL detection kit (Pierce). A comparative analysis was performed using quantitative densitometry.

### ELISA

The concentrations of IFN-γ, IL-4, IL-17 and insulin in CD4^+^ T-cell culture media or mouse serum were measured by enzyme-linked immunosorbent assay (ELISA) according to the manufacturer's instructions (cytokines, R&D Systems; insulin, LINCO Research, Inc., St. Charles, MO). All assays were performed in triplicate.

### Statistics

Data were expressed as mean ± SEM. One-way analysis of variance (ANOVA), followed by post-hoc SNK test, was used for comparisons between groups. *p* < 0.05 was considered statistically significant.

## Supplementary material

Detailed facts of importance to specialist readers are published as ”Supporting Information”. Such documents are peer-reviewed, but not copy-edited or typeset. They are made available as submitted by the authors.
