# Supplementary material for: A guanidine-rich regulatory oligodeoxynucleotide improves type-2 diabetes in obese mice by blocking T-cell differentiation
Source: EMBO Mol Med. 2012 Oct 2;4(10):1112–25. doi: 10.1002/emmm.201201272 (PMC3491840; doi:10.1002/emmm.201201272)
Supplement: Supplementary file 1 [file emmm0004-1112-SD1.pdf]

EMBO
Molecular Medicine

Manuscript EMM-2012-01272

A guanidine-rich regulatory oligodeoxynucleotide improves type-2 diabetes in obese mice by blocking T-cell differentiation

Xiang Cheng, Jing Wang, Ni Xia, Xin-Xin Yan, Ting-Ting Tang, Han Chen, Hong-Jian Zhang, Juan Liu, Wen Kong, Sara Sjˆberg, Eduardo Folco, Peter Libby,Yu-Hua Liao, Guo-Ping Shi

Corresponding author: Guo-Ping Shi, Brigham and Women's Hospital

Review timeline:

Submission date:	09 February 2012
Editorial Decision:	13 March 2012
Revision received:	29 May 2012
Editorial Decision:	14 June 2012
Revision received:	01 August 2012
Accepted:	08 August 2012

Transaction Report:

(Note: With the exception of the correction of typographical or spelling errors that could be a source of ambiguity, letters and reports are not edited. The original formatting of letters and referee reports may not be reflected in this compilation.)

1st Editorial Decision 13 March 2012

Thank you for the submission of your manuscript "A guanidine-rich regulatory oligodeoxynucleotide improves type-2 diabetes by blocking T-cell differentiation" to EMBO Molecular Medicine.

We have now heard back from the referees whom we asked to evaluate your manuscript. You will see that they find the topic of your manuscript potentially interesting. However, they also raise significant concerns on the study, which should be addressed in a major revision of the manuscript.

In particular, reviewers #1 and reviewer #2 highlight that the adipocyte size reduction should be further investigated. In addition, Reviewer #1 notes concerns about the macrophage experiments.

Given the balance of these evaluations, we feel that we can consider a revision of your manuscript if you can convincingly address the issues that have been raised within the space and time constraints outlined below.

Revised manuscripts should be submitted within three months of a request for revision. They will otherwise be treated as new submissions, unless arranged otherwise with the editor.

I look forward to seeing a revised form of your manuscript as soon as possible.

Yours sincerely,

Editor
EMBO Molecular Medicine

***** Reviewer's comments *****

Referee #1:

Shorita's group and others previously showed that synthetic ODNs containing TTAGGG motifs exert regulatory effects in vivo, by means of dampening Th1-mediated responses via inhibition of STAT3/4 phosphorylation and signaling. In the present work, Cheng et al. synthesized novel regulatory ODNs modifying the lead, ODNA151, and introducing new deoxynucleotides in the sequence upstream the poly(G) motif. They found novel immunoregulatory ODNs, among which ODNR01 was identified as the most potent. ODNR01 inhibited STAT3/4 and also STAT1 but not STAT5/6 phosphorylation. In addition, the same ODN did not interfere with c-Jun N-terminal kinase, MAPK p38 and NF-kB (data not shown). Based on the rationale that Th1 cells play a major role in driving T2D and obesity, ODNR01, after preliminary studies in healthy, Th1-prone C57BL6 and Th2-prone Balb/c mice (in which the ODN was demonstrated to inhibit Th1 and Th17 but not Th2 and Treg development), was used to treat mice affected by T2D and obesity, i.e. ob/ob and DIO mice. They found that ODNR01 did not reduce body weight and did not significantly modify serum total cholesterol, HDL and triglyceride levels. However, it did reduce visceral and subcutaneous fat mass, adipocyte size, fasting insulin levels and improved glucose tolerance and insulin sensitivity. In the inflammatory side of the disease, ODNR01 reduced the percentages of Th1 and Th17 but did not modify the percentages of Th2 and Treg cells both locally (SVF) and in the spleen. Furthermore, ODNR01 reduced M1 cell accumulation in adipose tissues but it appears that the agent, though colocalizing with STAT1/3/4 in macrophages, does not inhibit cytokine production in these cells. Experiments with Rag1-/- mice, including adoptive cell transfer experiments with CD4+ T cells, further suggested that the immunoregulatory effects ODNR01 are mediated by T cells. Although the use of immunoregulatory ODNs may be of interest for the treatment of T2D/obesity, I have some concerns that preclude the publication of the manuscript in its current form.

1. The Authors synthesized novel ODNs, although ODNA151 has already been demonstrated to be effective. So the rationale of using their own ODNs would be correct if they prove that the new ones are more effective than the old one. However, I do not see any statistical comparison between ODNA151 and ODNR01 effects (only compared in Fig. 1). It might also be important to compare them again in some other experiments, particularly those aimed at analyzing molecular mechanisms. Furthermore, "old" ODNs have been more extensively characterized. Do the "new" ODNs require TLR9?

2. Fig. 1D/E: How were the percentages of Th cells measured? From the previous part of the Fig., it might be deduced that this is in term of cytokine-producing cells and not true Th1 or not Th17 cells. Percentages of Th subset should be measured by costaining of CD4 and Tbet or RORgammat.

3. The Authors should at least discuss how the discrepancy between lack of inhibition of body

weight and reduction of fat body mass may be reconciled.

4. The important effect of adipocyte size reduction requires analysis of adiponectin production, know to be crucial in such effect.

4. Authors cannot speak of a Th2 and Treg dominance (see for instance bottom of page 9), since there is inhibition of Th1/Th17 and no effect on the former subsets. They should speak rather of a shift in the balance.

5. Role of macrophages: although Rag1-/- and cell transfer experiments would indicate T cells as the most important mediators of ODNR01, the experiments conducted with macrophages are not conclusive. In fact, Authors should repeat their experiments in conditions of macrophage activation, i.e. after stimulation with IFN-g and therefore in conditions where phosphorylation of STATs (i.e. STAT1) could be observed (as has been done for T cells).

Referee #2:

This is the comprehensive study of the effect of ODNR on immune cells, and diabetic and obese conditions. However, there lack the biological significance of this nucleotide.

1) This nucleotides have some physiological meanings associated with obesity? If not, the scientific strength of this manuscript would be limited.

2) Why ODNR001 treatment restrict the adipocyte hypertrophy without changing the food intakes. Metabolic parameters including oxygen consumption, and serum cytokines should be examined to elucidate the systemic effect of these nucleotides.

3) What is the clinical significant of these nucleotides. Oral administration can be performed similarly?

4) FACS analysis of M1, and M2 macrophages in VAT should be performed in ODNR treated animals, not by immuno-staining.

5) Detailed experiment protocols should be described in figure legends. For example, how the T cells are administered to animals in figure 4D? What time? How?

6) It is hard to understand the meaning of Fig 1D for general readers. What the X-axis stand for? This reviewer recommends the use of quantification graphs in place of representative FACS double plot.

7) The same in Fig 4A. If gated CD4+ T cells, why the X-axis need to stand for CD4 signals?

8) In figure2 legends, the cell types used should be described in details. Not only "T cells".

9) There are no needs to use color panels in figs 3, and 4.

1st Revision - Authors' Response 29 May 2012

Point-by-Point Responses

Referee #1:

We thank this reviewer for his/her time and effort in evaluating our manuscript, and for the insightful comments that have greatly improved our revision. ***The reviewer's original comments are cited in bold and italic,*** followed by our responses. New text is underlined.

1. The Authors synthesized novel ODNs, although ODNA151 has already been demonstrated to be effective. So the rationale of using their own ODNs would be correct if they prove that the new ones are more effective than the old one. However, I do not see any statistical comparison between ODNA151 and ODNR01 effects (only compared in Fig. 1). It might also be important to compare them again in some other experiments, particularly those aimed at analyzing molecular mechanisms. Furthermore, "old" ODNs have been more extensively characterized. Do the "new" ODNs require TLR9?

We thank the reviewer for these comments and suggestions. In Figure 1A, we compared ODNA151 with our four new ODNs and control ODN1612 in regulating different cytokine expressions from $CD4^+$ T cells under different culture conditions.

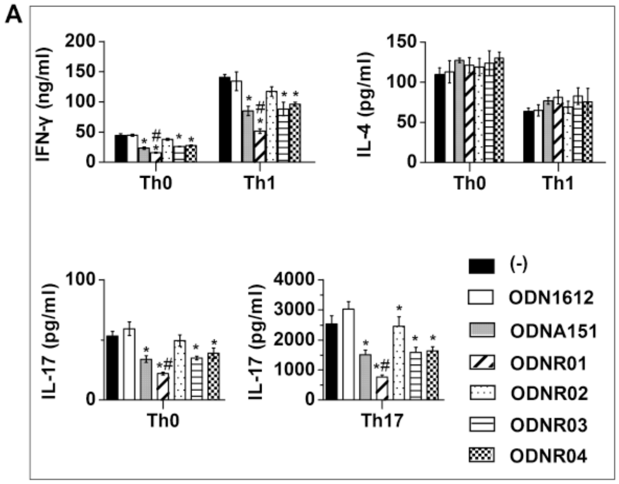

Figure 1A

At the reviewer's suggestion, we compared ODNR01 with ODNA151 and ODN1612 on Th1 and Th17 cell differentiation from Th1-biased C57BL/6 mice. We have incorporated these data into the revised Supplementary Figure 1A/1B, and have discussed them in the text on page 6, line 18 to page 7, line 1.

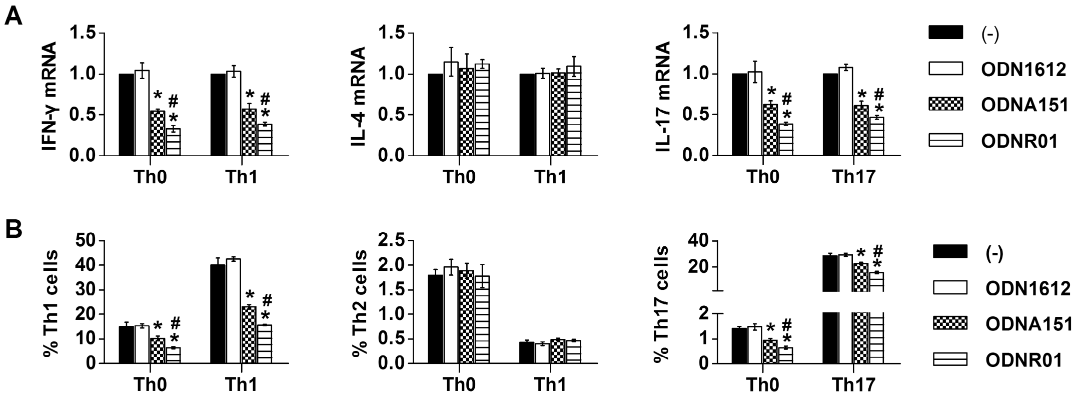

Supplementary Figure 1A/1B

As reported by Shirota H, et al. (J Immunol. 2004;173:5002-7), the activities (e.g., binding to STAT1/4 and blocking their phosphorylation) of this class of regulatory ODNs (e.g., ODN151) do not require the involvement of TLR9. We have added this sentence to the revised text on page 4, lines 8-11.

Page 6, line 18 to page 7, line 1: Under the same concentration (5 μM), ODNR01 was significantly stronger than ODNA151 in inhibiting the expression of IFN-γ and IL-17 or Th1 and Th17 cell frequencies, but had no inhibitory effect on IL-4 expression or Th2 cell frequencies in peripheral CD4$^+$ T cells from Th1-biased C57BL/6 mice, while the control ODN1612 had no inhibitory activities (Supplementary Figure 1A/1B).

Page 4, lines 8-11: In contrast, *regulatory* ODNs act by selectively binding to STAT1 and STAT4, then blocking their subsequent phosphorylation — and these activities do not require binding to receptors such as TLR9 (Shirota et al, 2004; Shirota et al, 2005).

2. Fig. 1D/E: How were the percentages of Th cells measured? From the previous part of the Fig., it might be deduced that this is in term of cytokine-producing cells and not true Th1 or not Th17 cells. Percentages of Th subset should be measured by costaining of CD4 and Tbet or RORgammat.

We agree fully with the reviewer. In Fig.1D/E, purified CD4$^+$ T cells were polarized under Th0, Th1, or Th17 conditions in the absence or presence of ODNs. After a 4-day culture, cells were re-stimulated with phorbol 12-myristate 13-acetate (PMA) and ionomycin in the presence of GolgiPlug, collected, and then stained intracellularly with anti-IFN-γ, anti-IL-4, and anti-IL-17 antibodies. Because the cells were sorted CD4$^+$ T cells, we omitted the surface staining of CD4. The percentage of IFN-γ$^+$, IL-4$^+$, and IL-17$^+$ cells in the collected cells (pre-sorted CD4$^+$ T cells) analyzed by FACS represent the Th1, Th2, and Th17 cell population (*J Biol Chem* 2009;284:28420; *Nature* 2008;454:350). To avoid the misunderstanding pointed out by the reviewer, we have changed the dot plots into

histograms in the revised Fig. 1D, and the figure legend has been revised accordingly. We have revised the text on page 6, lines 9-18 to reflect the changes.

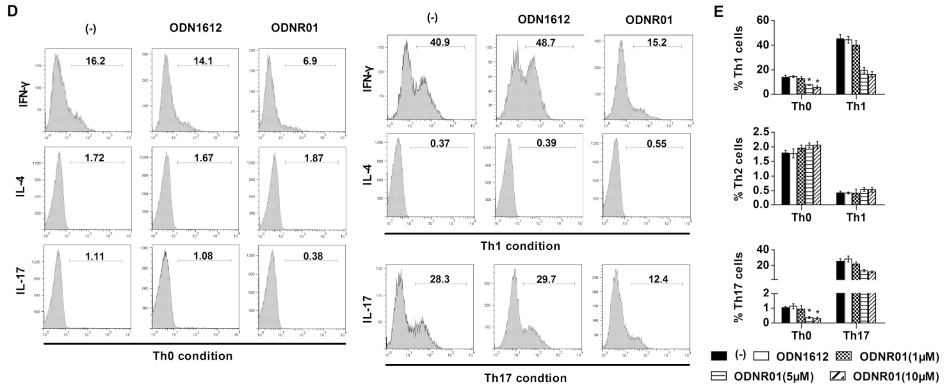

Figure 1D/1E

Page 6, lines 9-18: Flow cytometry histogram analysis further demonstrated a role of ODNR01 in inhibiting Th1 and Th17 cell differentiation, but not Th2 or Treg differentiation. When purified $CD4^+$ T cells were cultured under Th0, Th1, and Th17 conditions, followed by intracellular staining with anti-IFN-γ, anti-IL-4, and anti-IL-17 antibodies to detect Th1, Th2, and Th17 cell populations (Cui et al, 2009; Yang et al. 2008), we found that under Th0 conditions, 5~10 μM of ODNR01 reduced the percentage of Th1 and Th17 cells. Under Th1 and Th17 conditions, 5~10 μM of ODNR01 inhibited the percentages of Th1 cells and Th17 cells significantly (Figures 1D and 1E). ODNR01 at any tested concentration, however, did not affect Th2 cells (Figures 1D and 1E) or $CD4^+CD25^+Foxp3^+$ Treg frequency (data not shown).

3. The Authors should at least discuss how the discrepancy between lack of inhibition of body weight and reduction of fat body mass may be reconciled.

As shown in the following table and Figure 3B, VAT and SAT fat mass from ob/ob and DIO mice weighed about 0.3-gram less in ODNR01-treated mice than in PBS- or ODN1612-treated mice. The reduction of fat mass is negligible relative to the whole body weight, which accounted for the insignificant differences in body weight gain between the groups. We have discussed these observations in the revised text on page 9, lines 12-16.

Table. VAT and SAT fat mass in ob/ob and DIO mice among different treatments.

	ob/ob		DIO	
	VAT (g)	SAT (g)	VAT (g)	SAT (g)
PBS	2.86±0.0953	2.38±0.0947	2.04±0.0644	1.54±0.0429
ODN1612	2.80±0.0616	2.34±0.0666	2.03±0.0658	1.58±0.0542
ODNR01	2.56±0.0579*	2.06±0.0806*	1.82±0.0386*	1.34±0.0344**

Values are mean±SEM. N=7~8. *$P<0.05$, **$P<0.01$ versus respective PBS group.

Page 9, lines 12-16: Both visceral adipose tissue (VAT) and subcutaneous adipose tissue (SAT) fat mass, however, weighed about 0.3-gram less in ODNR01-treated mice than in PBS-treated mice in both the *ob/ob* and DIO groups (Figure 3B). ODNR01-induced reductions in fat mass did not lead to significant changes in body weight gain from those of PBS- or ODN1612-treated mice (Figure 3A).

4. The important effect of adipocyte size reduction requires analysis of adiponectin production, know to be crucial in such effect.

We thank the reviewer for raising this interesting concern. In humans, adipocyte sizes may positively (*J Clin Endocrinol Metab.* 2007;92:1023) or negatively (*J Endocrinol Invest.* 2007;30:210) associate with plasma adiponectin levels. To assess whether reduced adipocyte sizes in ODNR01-treated mice also affect adiponectin levels, we performed adiponectin ELISA and found no statistically significant differences from those in PBS- or control ODN1612-treated mice (see Figure below). These data are consistent with insignificant differences in body weight gain among the same groups of mice. We have discussed these results in the revised text on page 9, lines 19-21.

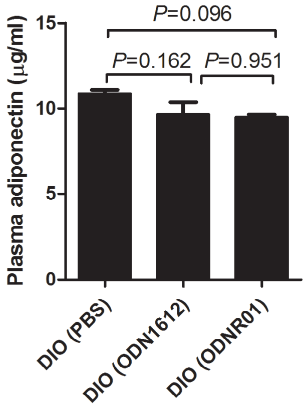

Page 9, lines 19-21: Adipocyte size often associates with plasma adiponectin production (Skurk et al., 2007; Bahceci et al., 2007), but in our study, ODNRO1-induced adipocyte size changes did not affect plasma adiponectin levels (data not shown).

5. Authors cannot speak of a Th2 and Treg dominance (see for instance bottom of page 9), since there is inhibition of Th1/Th17 and no effect on the former subsets. They should speak rather of a shift in the balance.

We thank the reviewer for this comment. We have corrected our statements in the revised text on page 10, lines 9-12, and page 16, lines 7-8.

Page 10, lines 9-12: ODNR01 effectively inhibited Th1 and Th17 cell differentiation *in vitro* (Figures 1 and 2), leading to a shift in balance toward Th2 and Treg and improved insulin sensitivity in obese mice (Figures 3D and 3E).

Page 16, lines 7-8: The current study used ODNR01 to regulate the CD4^{+} T-cell balance by blocking Th1 and Th17 cell differentiation.

6. Role of macrophages: although Rag1-/- and cell transfer experiments would indicate T cells as the most important mediators of ODNR01, the experiments conducted with macrophages are not conclusive. In fact, Authors should repeat their experiments in conditions of macrophage activation, i.e. after stimulation with IFN-g and therefore in conditions where phosphorylation of STATs (i.e. STAT1) could be observed (as has been done for T cells).

We appreciate this insightful comment. In Figures 5B and 5C, we showed that ODN01 inhibited the expression of M1 markers, but had no effect on M2 markers, in VAT. In contrast, ODNR01 showed no effect on either M1 or M2 markers when bone marrow-derived macrophages (BMDM) were used in vitro (Figure 5D). Therefore, we hypothesized that the presence of T cells, especially Th1 cells or Th1 cytokines, is essential for ODNR01 to show its activities. As the reviewer suggested, we cultured the same BMDMs in the presence of Th1 cytokine IFN-γ. Under the stimulation with this cytokine, ODNR01 showed its significant inhibitory effect on macrophage M1 marker expression, but remained ineffective on macrophage M2 marker expression — similar to what we observed in VAT (Figure 5B/5C). We have incorporated these new data to the revised Figure 5E, and have discussed these observations on page 12, lines 14-20.

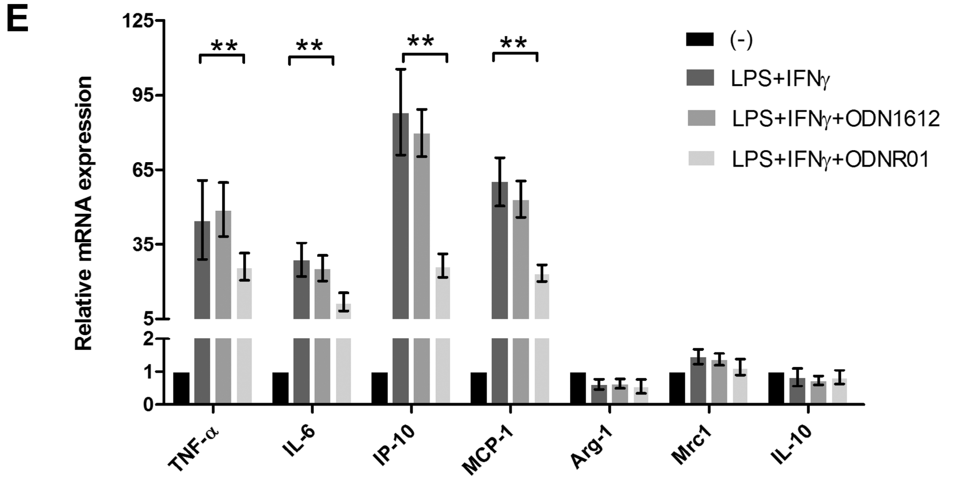

Figure 5E

Page 12, lines 14-20: Therefore, ODNR01 did not play a direct role in M1 cell recruitment, polarization, or activation in WAT, but instead participated via other cells or associated molecules (e.g., Th1 cells or Th1 cytokines). To test this hypothesis, we performed the same experiments as in Figure 5D. Instead of using naïve BMDMs, we activated BMDMs with lipopolysaccharide (LPS, 100 ng/mL) and Th1 cytokine IFN-γ (100 U/mL) (Khallou-Laschet et al, 2010). Under these conditions, ODNR01 showed significant inhibitory effects on M1 marker expression, but remained ineffective on M2 marker expression (Figure 5E).

Referee #2:

We thank this reviewer for his/her time and effort in evaluating our original submission. We appreciate his/her belief that our study "is the comprehensive study of the effect of ODNR on immune cells, and diabetic and obese conditions." ***The reviewer's original critiques are highlighted in bold and italic,*** followed by our responses. New text is underlined.

1) This nucleotides have some physiological meanings associated with obesity? If not, the scientific strength of this manuscript would be limited.

Although additional mechanisms may exist, as discussed in this study, regulatory ODNs regulate T-cell polarization and consequent M1 macrophage activities — all of which associate with obesity and diabetes.

2) Why ODNR001 treatment restrict the adipocyte hypertrophy without changing the food intakes. Metabolic parameters including oxygen consumption, and serum cytokines should be examined to elucidate the systemic effect of these nucleotides.

This study revealed a role of ODN in inhibiting Th1 and Th17 cells, but not Th2 and Treg cells (e.g., Figure 4). As we discussed on page 17, lines 4-8, for unknown reasons, Th1 cytokines (IFN-γ) affect adipocyte size, adipose tissue inflammation, and glucose sensitivity, but do not affect body weight gain or food intake (Rocha et al., *Circ Res* 2008;103:467). This reviewer's comments have been well taken, however, and as suggested, we measured the plasma adiponectin concentrations from DIO (PBS), DIO (ODN1612), and DIO (ODNR01) groups. We found no significant differences between the groups (see Figure below). We have discussed these data in the revised text on page 9, lines 19-21.

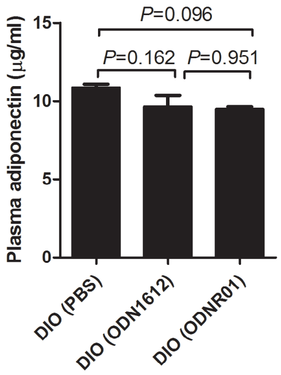

Page 17, lines 4-8: In DIO mice, the absence of Th1 cytokine IFN-g led to reductions in WAT macrophages, adipocyte size, and expression of inflammatory molecules (TNF-α and MCP-1). These mice demonstrated increased glucose sensitivity but showed no difference in body weight gain compared with WT control mice (Rocha et al, 2008), suggesting a negligible role of Th1 cells in controlling body weight.

Page 9, lines 19-21: Adipocyte size often associates with plasma adiponectin production (Skurk et al., 2007; Bahceci et al., 2007), but in our study, ODNRO1-induced adipocyte size changes did not affect plasma adiponectin levels (data not shown).

3) What is the clinical significant of these nucleotides. Oral administration can be performed similarly?

Although these nucleotides may have therapeutic potential (*Ann N Y Acad Sci.* 2009;1175:80), whether oral administration will yield effects similar to intraperitoneal injections in T-cell activity regulation remains untested. To avoid reader confusion, we have only briefly discussed this potential on page 19, lines 14-17.

Page 19, lines 14-17: These results affirm the regulatory role of adaptive immunity in the metabolic disorders associated with obesity, and present a potential therapeutic avenue for patients (Klinman et al., 2009) with, or predisposed to, type-2 diabetes — although orally available ODNs have not been tested.

4) FACS analysis of M1, and M2 macrophages in VAT should be performed in ODNR treated animals, not by immuno-staining.

We agree with the reviewer. It would have been reasonable to perform FACS analysis when we harvested these mice to detect M1 and M2 cells in VAT, rather than just immunostaining. As the reviewer suggests, we have performed RT-PCR to determine the mRNA levels of M1 and M2 markers in VAT. We present these data in the revised Figure 5B/5C.

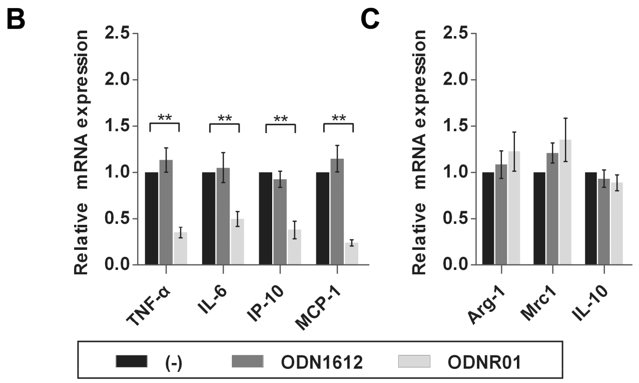

Figure 5B/5C

5) Detailed experiment protocols should be described in figure legends. For example, how the T cells are administered to animals in figure 4D? What time? How?

As suggested, we have added detailed experiment protocols to the figure legends — for example, Figure 6B.

Figure 6B. FACS plots to detect $CD3^+CD4^+$ T cells in VAT, SAT, and spleen from *Rag1*$^{-/-}$ mice that consumed HFD for 18 weeks (no ODN treatments), then received WT naive $CD4^+$ T-cell intraperitoneal transfer (5×10^6/mouse) at week 26, and consumed HFD for an additional 4 weeks while receiving ODN treatments.

6) It is hard to understand the meaning of Fig 1D for general readers. What the X-axis stand for? This reviewer recommends the use of quantification graphs in place of representative FACS double plot.

At the reviewer's suggestion, we have changed the dot plots into histograms in the revised Figure 1D. We also believe that histograms are much easier to read than the dot plots.

7) The same in Fig 4A. If gated CD4+ T cells, why the X-axis need to stand for CD4 signals?

As suggested and to avoid confusion, we have labeled the X-axis as "CD4 (gated cells)" in the revised Figure 4A, which was used previously by Winer et al. (Figure 4a, *Nat Med* 2009;15:921).

8). In figure2 legends, the cell types used should be described in details. Not only "T cells".

We thank the reviewer for this correction. In the revised Figure 2 legend, we have specifically mentioned that $CD4^+$ T cells were used.

9) There are no needs to use color panels in figs 3, and 4.

We thank the reviewer for this suggestion. We have changed Figures 1 and 4 to black-and-white, but we believe that Figure 3 would be best presented in color.

2nd Editorial Decision 14 June 2012

Thank you for the submission of your revised manuscript "A guanidine-rich regulatory oligodeoxynucleotide improves type-2 diabetes by blocking T-cell differentiation" to EMBO Molecular Medicine. We have now received the enclosed report from the referee that was asked to re-assess it.

As you will see, the reviewer still raises concerns that should be convincingly addressed. While we agree with the reviewer that further insight into the molecular mechanism of the ODNs would strengthen the manuscript and we encourage you to include respective data if you have generated them, we acknowledge the potential translational aspects of the study. As such, we would not consider the addition of more mechanistic data compulsory for the potential acceptance of the manuscript. However, the reviewer still raises the concern regarding the clear demonstration of Th1/Th17 differentiation. Since this is a major point of the paper, it will be crucial to experimentally address this concern. In addition, the reviewer raises a concern regarding the effect of the new ODN on activated macrophage, which should be convincingly addressed. Since we do acknowledge the potential interest of your findings, we would therefore be open to allow a second revision of the manuscript that would address the outstanding issues.

On a more editorial note, please also address the following points (Please see our Author Guidelines for more information):
- The description of all reported data that includes statistical testing must state the name of the statistical test used to generate error bars and P values, the number (n) of independent experiments underlying each data point (not replicate measures of one sample), and the actual P value for each test (not merely 'significant' or 'P < 0.05').
- EMBO Molecular Medicine does not permit citation of "Data not shown". All data referred to in the paper should be displayed in the main or supporting figures.
- The section 'For More Information' is intended to list relevant web links for further consultation by our readers, not related articles. Examples for relevant web links include patient associations, relevant databases, OMIM/proteins/genes links or author's websites.

Revised manuscripts should be submitted within three months of a request for revision; they will otherwise be treated as new submissions, unless arranged otherwise with the editor.

I look forward to seeing a revised form of your manuscript as soon as possible.

Yours sincerely,

Editor
EMBO Molecular Medicine

***** Reviewer's comments *****

Referee #1:

Authors have "apparently" answered to all the questions raised by this reeferee. I say apparently because they thanked for the comments and suggestions but did not answered consequently. Specifically, they continued to use the terminology Th1 and Th17 just by virtue of the T cell capacity of producing either IFN-gamma or IL-17, but did not perform any experiment showing whether these cells are Tbet+ and Rorgt+, respectively. In this regard, they referred to two specific publications (authors, titles?), which I tried to search in the specific journals without success...

Furthermore, they did not perform any experiments aimed at evaluating something related to molecular mechanism of their new ODNs that I instead think would be necessary for a journal such as EMBO. They did perform the experiment, as suggested by this referee, with macrophages activated by IFN-gamma and they did find that that the ODN treatment impairs the cytokine response of these cells. However, they continue to say that all the effect is mediated by T cells,

whereas the experiment with activated macrophages suggest something else. Thus I think that both technical and theoretical aspects in this paper are still insufficient for publication in EMBO.

2nd Revision - Authors' Response 01 August 2012

Reviewer #1

We thank this reviewer for his/her time in evaluating our prior two submissions. His/her remaining concerns, which further improved this revision, are cited in *italic*, followed by our responses. New text is underlined.

Specifically, they continued to use the terminology Th1 and Th17 just by virtue of the T cell capacity of producing either IFN-gamma or IL-17, but did not perform any experiment showing whether these cells are Tbet+ and Rorgt+, respectively. In this regard, they referred to two specific publications (authors, titles?), which I tried to search in the specific journals without success...

As suggested by the reviewer, we performed FACS analysis to detect T-bet+ and Rorgt+ cells under Th0, Th1, and Th17 conditions. We have presented these new data in the revised Figure 1D and 1E, and have discussed them in the revised Results section on page 6, lines 9-21.

Figure 1 Legend: D. Representative FACS histograms of Th1 and Th17 cells for purified $CD4^+$ T cells treated under Th0, Th1, or Th17 conditions with or without ODN (5 μM) for 4 days. Lymphocytes were first gated on the SSC/FSC plots, and then the expression of T-bet and RORγt on the purified $CD4^+$ T cells was analysed. E. Th1 or Th17 cell frequencies in $CD4^+$ T cells after cells were treated with ODNs as in B for 4 days, **P*<0.05 *vs.* (-) and ODN1612. Data are representative of three independent experiments.

Page 6, lines 9-21: Flow cytometry histogram analysis further showed that ODNR01 inhibits Th1 and Th17 cell differentiation, but not Th2 or Treg differentiation. When purified $CD4^+$ T cells were cultured under Th0, Th1, and Th17 conditions, followed by intracellular staining with anti-T-bet and anti- RORγt antibodies to detect Th1 and Th17 cell populations (Cui et al, 2009; Yang et al. 2008), we found that under Th0 conditions, 5~10 μM of ODNR01 reduced the percentage of Th1 and Th17 cells. Under Th1 and Th17 conditions, 5~10 μM of ODNR01 inhibited the percentages of Th1 cells and Th17 cells significantly (Figures 1D and 1E). ODNR01 at any tested concentration, however, did not affect Th2 cells or $CD4^+CD25^+Foxp3^+$ Treg frequency (Supplementary Figure 1). In the same concentration (5 μM), ODNR01 was significantly stronger than ODNA151 in inhibiting the expression of IFN-γ and IL-17 or Th1 and Th17 cell frequencies, but had no inhibitory effect on IL-4 expression or Th2 cell frequencies in peripheral $CD4^+$ T cells from Th1-biased C57BL/6 mice, while the control ODN1612 had no inhibitory activity (Supplementary Figures 2A and 2B).

2. Furthermore, they did not perform any experiments aimed at evaluating something related to molecular mechanism of their new ODNs that I instead think would be necessary for a journal such as EMBO.

In the revised Figure 2A and 2B, we have added new data to compare ODNR01 with the known ODNA151 in STAT1/3/4/5/6 phosphorylation. Our new data demonstrated that ODNR01

suppressed STAT1/3/4 phosphorylation more potently than ODNA151 did. We have revised the Methods section on page 21, lines 14-18, and have discussed these data on page 7, lines 13-22.

Figure 2 A-B legend: A/B. STAT and phospho-STAT Western blot analysis of anti-CD3/28 mAb-stimulated $CD4^+$ T cells treated with indicated cytokines, with and without 5 μM of ODNR01, ODNA151, or ODN1612.

Page 21, lines 14-18: To study the effects of ODNs on $CD4^+$ T-cell differentiation signal pathways, anti-CD3/28 mAb-stimulated $CD4^+$ T cells were incubated with different stimuli (e.g., IFN-γ, IL-12, IL-2, and IL-4), washed with PBS, and lysed in ice-cold PhosStop lysis buffer containing protease and phosphatase inhibitors (Roche Applied Science, Indianapolis, IN). Protein extracts were used for Western blot analysis.

Page 7, lines 11-20: While non-specific ODN1612 showed no effect on the phosphorylation of these STATs, 5 μM of ODNA151 and ODNR01 greatly inhibited the phosphorylation of STAT1 and STAT3/4 (Figure 2A). Under the same experimental conditions, ODNR01 appeared much more potent than ODNA151 in suppressing STAT1/3/4 phosphorylation (Figure 2A). IL-2 (10 ng/ml) and IL-4 (10 ng/ml) are commonly used to activate STAT5 and STAT6, which are critical to the differentiation of Treg and Th2, respectively (Burchill et al, 2007; Kaplan et al, 1996). Neither ODN1612 nor ODNA151 or ODNR01 inhibited STAT5 or STAT6 phosphorylation (Figure 2B), suggesting that ODNR01 — but not ODN1612 — inhibited the differentiation of Th1 and Th17 cells even more strongly than the known ODNA151, but not the differentiation of Treg or Th2 cells.

3. They did perform the experiment, as suggested by this referee, with macrophages activated by IFN-gamma and they did find that that the ODN treatment impairs the cytokine response of these cells. However, they continue to say that all the effect is mediated by T cells, whereas the experiment with activated macrophages suggest something else.

We thank the reviewer for this thoughtful comment, and we fully agree with it. Although ODNR01 did not affect M1 or M2 markers in naïve macrophages, it suppressed M1 markers, but not M2 markers, from activated macrophages (Figure 5E) and those from DIO mice (Figure 5B and 5C) and ob/ob mice (Supplementary Figure 7). We have discussed these data on page 12, lines 7-19.

Page 12, lines 7-19: Of note, when cultured naïve BMDMs were used, neither ODNR01 nor ODN1612 affected M1 or M2 cytokine and chemokine expression (Figure 5D), suggesting that ODNR01 affected macrophage polarization under certain conditions, such as in the presence of inflammation or under activation stimulation from other inflammatory cells or cytokines. To test this hypothesis, we performed the same experiments as in Figure 5D. Instead of using naïve BMDMs, we activated BMDMs with lipopolysaccharide (LPS, 100 ng/mL) and Th1 cytokine IFN-γ (100 U/mL) (Khallou-Laschet et al, 2010). Consistent with our hypothesis, under these conditions, ODNR01 showed significant inhibitory effects on M1 marker expression, but remained ineffective on M2 marker expression (Figure 5E). As in $CD4^+$ T cells (Figure 2C), ODNR01 but not ODN1612 also interacted with STAT1/3/4, but not with STAT5/6 in BMDMs. Confocal microscopy demonstrated that ODNR01 colocalized with STAT1/3/4, but not with STAT5/6 in BMDMs (Supplementary Figure 8). Therefore, ODNR01 targets not only T cells, but also macrophages under inflammatory conditions.
