# Supplementary material for: A guanidine-rich regulatory oligodeoxynucleotide improves type-2 diabetes in obese mice by blocking T-cell differentiation
Source: EMBO Mol Med. 2012 Oct 2;4(10):1112–25. doi: 10.1002/emmm.201201272 (PMC3491840; doi:10.1002/emmm.201201272)
Supplement: Supplementary file 2 [file emmm0004-1112-SD2.pdf]

TABLE OF CONTENTS:

**Table of contents**	**---**	**Page 1**
**Supplementary Figure 1**	**---**	**Page 2**
**Supplementary Figure 2**	**---**	**Page 3**
**Supplementary Figure 3**	**---**	**Page 4**
**Supplementary Figure 4**	**---**	**Page 5**
**Supplementary Figure 5**	**---**	**Page 5**
**Supplementary Figure 6**	**---**	**Page 6**
**Supplementary Figure 7**	**---**	**Page 6**
**Supplementary Figure 8**	**---**	**Page 7**
**Supplementary Figure 9**	**---**	**Page 8**

SUPPLEMENTARY MATERIALS

A guanidine-rich regulatory oligodeoxynucleotide improves type-2 diabetes in obese mice by blocking T-cell differentiation

Xiang Cheng, Jing Wang, Ni Xia, Xin-Xin Yan, Ting-Ting Tang, Han Chen, Hong-Jian Zhang, Juan Liu, Wen Kong, Sara Sjöberg, Eduardo Folco, Peter Libby, Yu-Hua Liao, Guo-Ping Shi

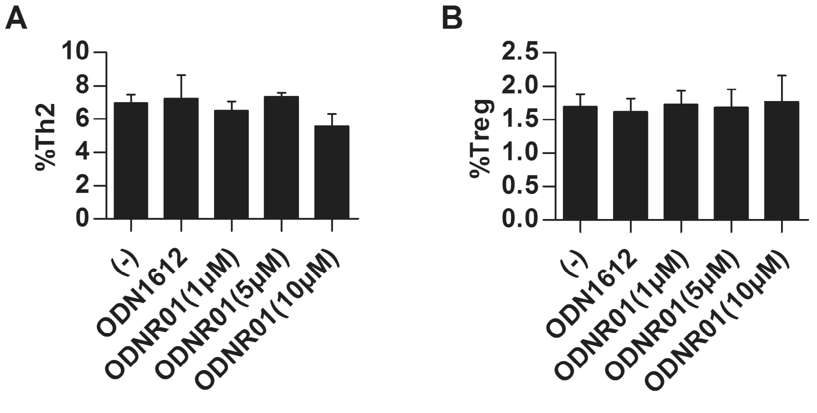

Supplementary Figure 1. Th2 (**A**) or Treg (**B**) cell frequencies in $CD4^+$ T cells after cells were treated with 5 μM ODN1612 or different amounts of ODNR01, as indicated, for 4 days. There were no significant differences among these treatments. Data are representative of three independent experiments.

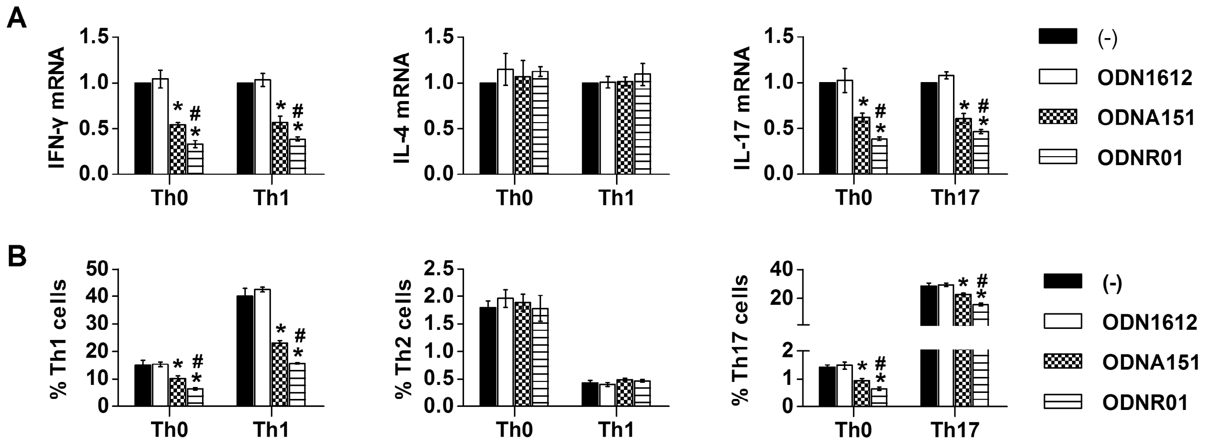

Supplementary Figure 2. Immunosuppressive activity of ODNR01 on Th1 and Th17 cell differentiation from Th1-biased C57BL/6 mice. **A.** $CD4^+$ T cells were treated with anti-CD3 and anti-CD28 mAb with or without (-) 5 µM different ODNs under Th0, Th1, or Th17 conditions for 3 days. IFN-γ, IL-4, and IL-17 mRNA levels were determined by RT-PCR. Immunosuppressive ODNA151 was used as a positive control, and random ODN1612 was used as a negative control. **B.** Th1, Th2, or Th17 cell frequencies in $CD4^+$ T cells after cells were treated with ODNs as in **A** for 4 days. Data represent three independent experiments. *P<0.05 *vs.* (-) and ODN1612; #P<0.05 *vs.* ODNA151.

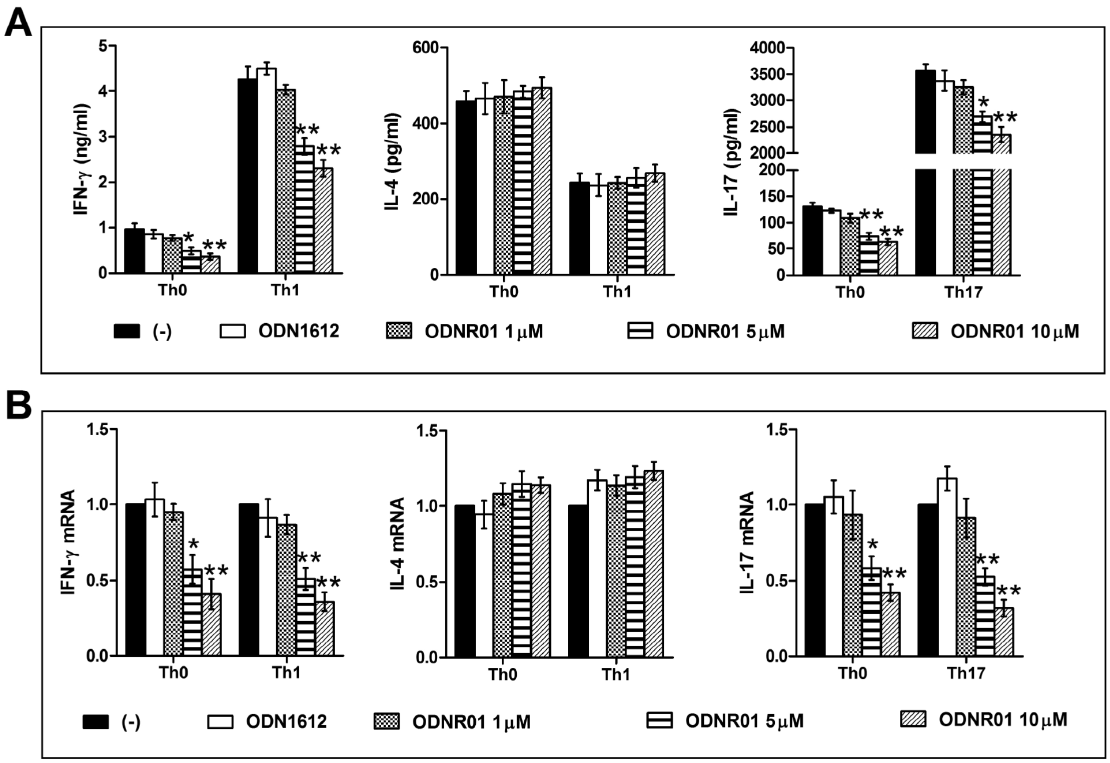

Supplementary Figure 3. Immunosuppressive activity of ODNR01 on Th1 and Th17 cell differentiation from Th2-biased Balb/c mice. **A.** $CD4^+$ T-cell culture media IFN-γ, IL-4, and IL-17 levels, as determined by ELISA. Splenic $CD4^+$ T cells from Balb/c mice were treated with anti-CD3 and anti-CD28 mAb with or without (-) ODN1612 (5 μM) or different doses of ODNR01 (1, 5, 10 μM) under Th0, Th1, or Th17 conditions. **B.** RT-PCR determined $CD4^+$ T-cell IFN-γ, IL-4, and IL-17 mRNA levels after cells were treated as in **A**. Data represent three independent experiments. **P*<0.05, ***P*<0.01 *vs.* (-) and ODN1612.

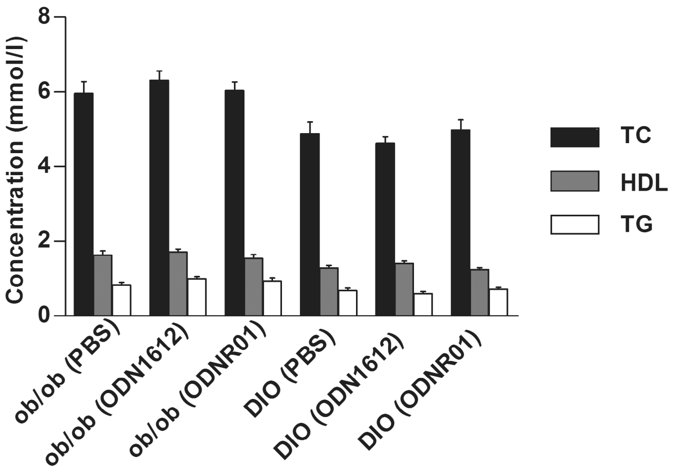

Supplementary Figure 4. ELISA determined mouse plasma total cholesterol (TC), high-density lipoprotein (HDL), and triglyceride (TG) levels in *ob/ob* mice (on a normal chow diet, NCD) and DIO mice (on a high-fat diet, HFD) receiving PBS, ODN1612, and ODNR01. There were no significant differences among the treatments. n=7~8 mice per group.

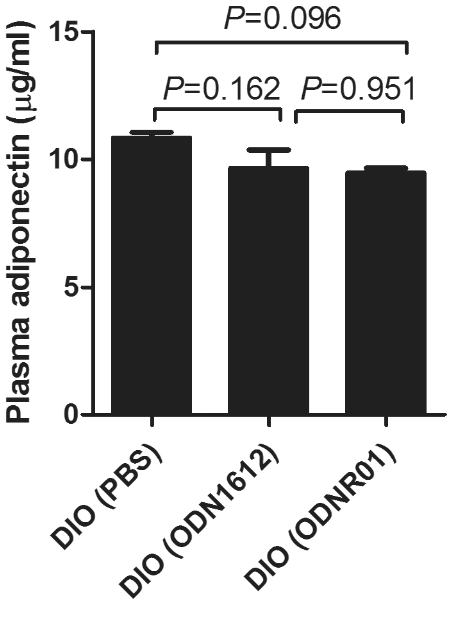

Supplementary Figure 5. ELISA determined mouse plasma adiponectin levels in DIO mice (on a high-fat diet, HFD) receiving PBS, ODN1612, and ODNR01. There were no significant differences among the treatments. n=7~8 mice per group.

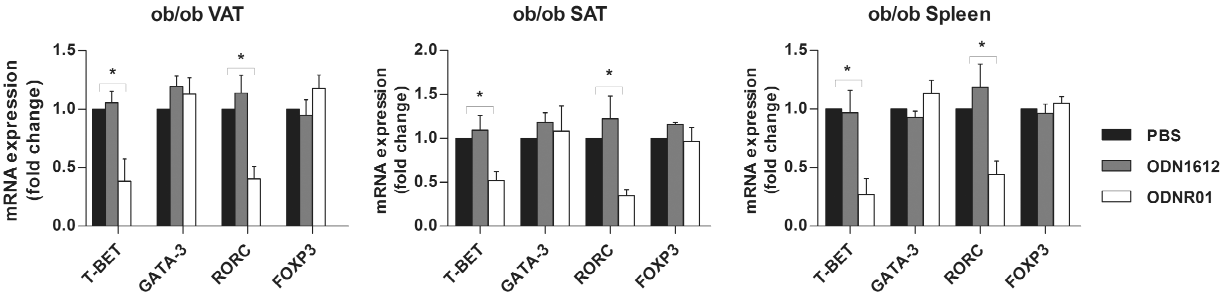

Supplementary Figure 6. RT-PCR determined mRNA levels of transcription regulators (T-bet, GATA-3, RORγt, and Foxp3) in sorted CD4^{+} T cells from VAT, SAT, and spleen of *ob/ob* mice treated with different ODNs. **P*<0.05, n=6~8 mice per group.

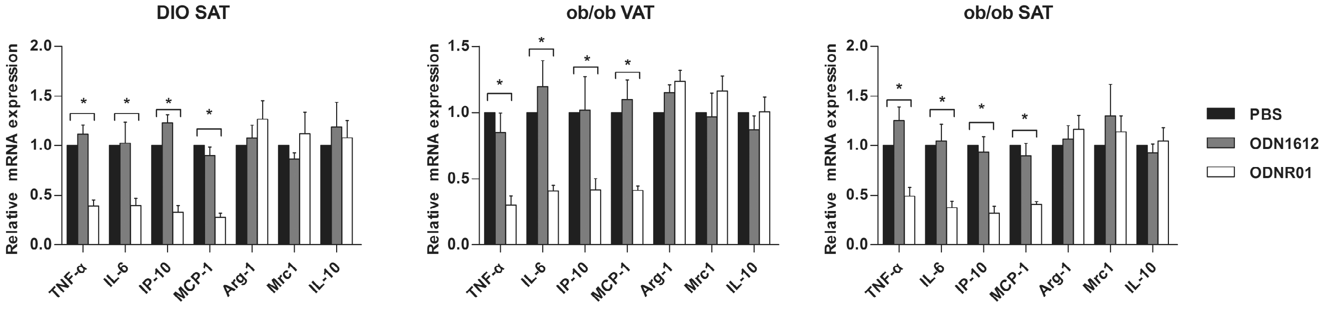

Supplementary Figure 7. RT-PCR determined mRNA levels of M1 markers (TNF-α, IL-6, IP-10, and MCP-1) and M2 markers (Arg1, Mrc1, and IL-10) in SAT from DIO mice and in VAT and SAT from *ob/ob* mice after normalizing to GAPDH. Fold change was calculated relative to PBS group. **P*<0.05; n=6 mice per group.

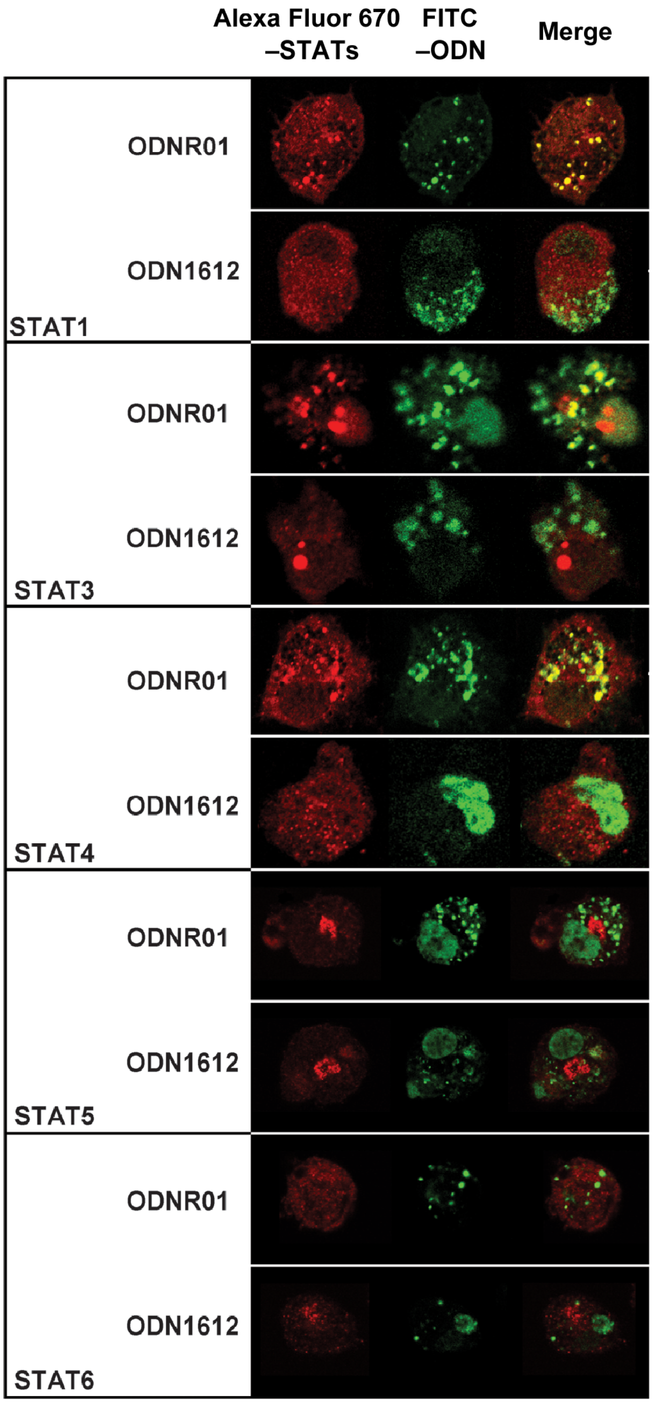

Supplementary Figure 8. Confocal microscopy to detect co-localization of FITC-conjugated ODNs (2.5 μM) (green) with different Alexa Fluor 670-conjugated anti-STAT rabbit polyclonal antibodies (red) in mouse bone marrow-derived macrophages after 24 hours of incubation.

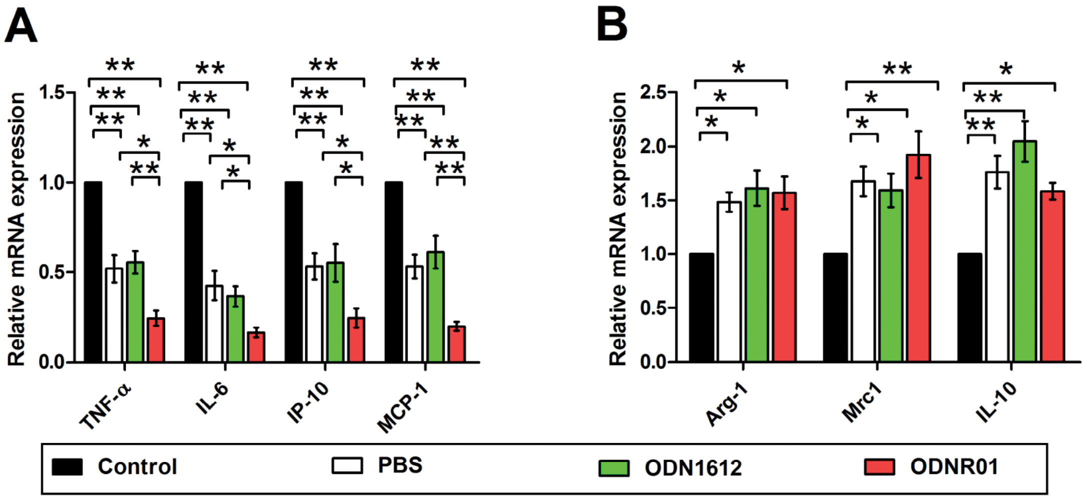

Supplementary Figure 9. RT-PCR determined mRNA levels of M1 markers (TNF-α, IL-6, IP-10, and MCP-1) (**A**) and M2 markers (Arg1, Mrc1, and IL-10) (**B**) in VAT after normalizing to GAPDH. Fold change was calculated relative to control group. Control: no reconstitution or treatment, but HFD for the same time period; PBS: $CD4^+$ T-cell-reconstituted mice were treated with PBS or mice reconstituted with PBS-treated $CD4^+$ T cells. *P<0.05, **P<0.01, n=6~8 mice per group.
